# Effects of small molecule-induced dimerization on the programmed death ligand 1 protein life cycle

**DOI:** 10.1038/s41598-022-25417-6

**Published:** 2022-12-09

**Authors:** Ilean Chai, Dmytro Kornyeyev, Edward Hsieh, Gesham Magombedze, Lance Stapleton, Magdeleine Hung, Hyock Joo Kwon, Erin Stefanutti, JeanPhilippe Belzile, Gregg Czerwieniec, Adele Y. Wang, Mariya Morar, Latesh Lad

**Affiliations:** Gilead Sciences, 333 Lakeside Drive, Foster City, CA 94403 USA

**Keywords:** Biochemistry, Cell biology, Oncology

## Abstract

The programmed death 1 (PD-1)/programmed death ligand 1 (PD-L1) checkpoint blockade is central to Immuno-Oncology based therapies, and alternatives to antibody blockers of this interaction are an active area of research due to antibody related toxicities. Recently, small molecule compounds that induce PD-L1 dimerization and occlusion of PD-1 binding site have been identified and developed for clinical trials. This mechanism invokes an oligomeric state of PD-L1 not observed in cells previously, as PD-L1 is generally believed to function as a monomer. Therefore, understanding the cellular lifecycle of the induced PD-L1 dimer is of keen interest. Our report describes a moderate but consistent increase in the PD-L1 rate of degradation observed upon protein dimerization as compared to the monomer counterpart. This subtle change, while not resolved by measuring total PD-L1 cellular levels by western blotting, triggered investigations of the overall protein distribution across various cellular compartments. We show that PD-L1 dimerization does not lead to rapid internalization of neither transfected nor endogenously expressed protein forms. Instead, evidence is presented that dimerization results in retention of PD-L1 intracellularly, which concomitantly correlates with its reduction on the cell surface. Therefore, the obtained data for the first time points to the ability of small molecules to induce dimerization of the newly synthesized PD-L1 in addition to the protein already present on the plasma membrane. Overall, this work serves to improve our understanding of this important target on a molecular level in order to guide advances in drug development.

## Introduction

Over the last decade, immune checkpoint blockade has emerged as a pillar of cancer immunotherapy^1,2^. A key checkpoint is the interaction between Programmed Death 1 (PD-1) protein and Programmed Death Ligand 1 (PD-L1) which leads to self-tolerance and results in tumors escape from immune surveillance^3,4^. Disruption of this interaction has proven clinically beneficial with seven monoclonal antibodies (mAb) currently approved by the US Food and Drug Administration and many more in development^1,5,6^.

However, while antibody therapies targeting PD-1/PD-L1 interaction have significantly advanced cancer management options, many patients fail to respond to this treatment in the long term, often for unclear reasons^1,7,8^. Hence deepening our understanding of cellular and molecular biology of this pathway, as well as uncovering novel inhibition modalities for the PD-1/PD-L1 checkpoint remain an important area of investigation.

**Figure 1:**
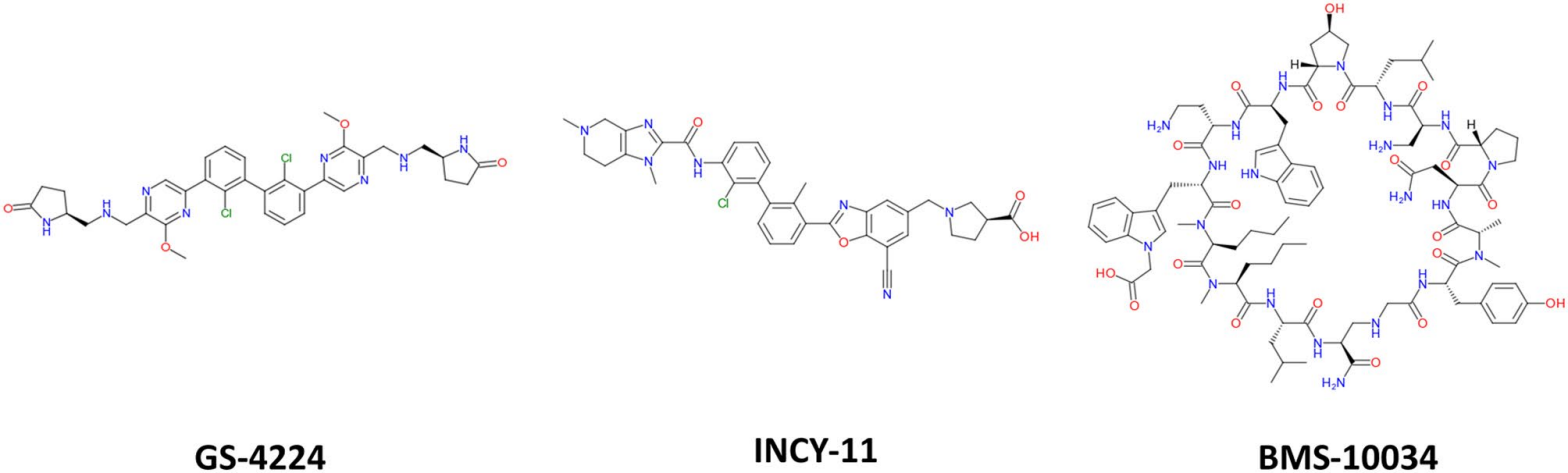
Chemical structures of compounds used in this study. GS-4224^27^ and INCY-11^26^ induce dimerization, while BMS-10034 is a non-dimerizing blocker^20^.

One alternative approach to inhibiting PD-1/PD-L1 interaction is through the development of a small molecule modulator. To date two major synthetic classes of small molecule inhibitors have been reported: peptide-based structures which serve to block the interaction site directly and multicyclic inhibitors which trigger PD-L1 dimerization and indirect occlusion of the PD-1 binding site^5,9–19^ (Fig. 1). These distinct mechanisms of inhibition have been extensively characterized in vitro with X-ray structures available for both modalities^9,20,21^. While oligomers of PD-L1 have been generated in vitro through fusions and in crystallization trials^22^, no evidence of cellular homodimerization has been reported to date for membrane-associated PD-L1. It is thus generally believed that this receptor acts as a monomer in checkpoint engagement^23^, and inhibitor-induced dimerization of PD-L1 represents a novel state for this protein. Recent publications investigating the consequences of dimerization have reported receptor internalization and degradation^24–26^. In this study we performed a detailed investigation of the PD-L1 dimer lifecycle at the cellular level: characterizing degradation kinetics, cellular localization and effects of dimerization on post-translational modifications. The results of the work show that dimerization affected the newly-synthesized intracellular PD-L1 protein in addition to the plasma membrane-localized receptor with minimal influence on internalization kinetics.

## Results

### PD-L1 relative degradation

The application of stable isotope labelling with amino acids in cell culture (SILAC) technology for pulse chase experiments to determine protein turnover and degradation rates has been demonstrated in multiple models^28–31^. In this study, the SILAC method was used to evaluate whether the inhibitors of PD-1/PD-L1 interaction, known to induce PD-L1 dimerization, influenced the rate of protein degradation. Previous reports have estimated that the half-life of PD-L1 is around 20 h (h)^32–35^. To more precisely determine the relative degradation rate of PD-L1, we chose to perform 48 h pulse chase experiments and apply a mathematical correction for each cell doubling event^36–38^. Such a model makes general assumptions about the steady state level of PD-L1 and cellular growth rate. However, it is a suitable approximation for the purpose of a direct comparison between equivalent samples subjected to different small molecule treatments.

**Figure 2:**
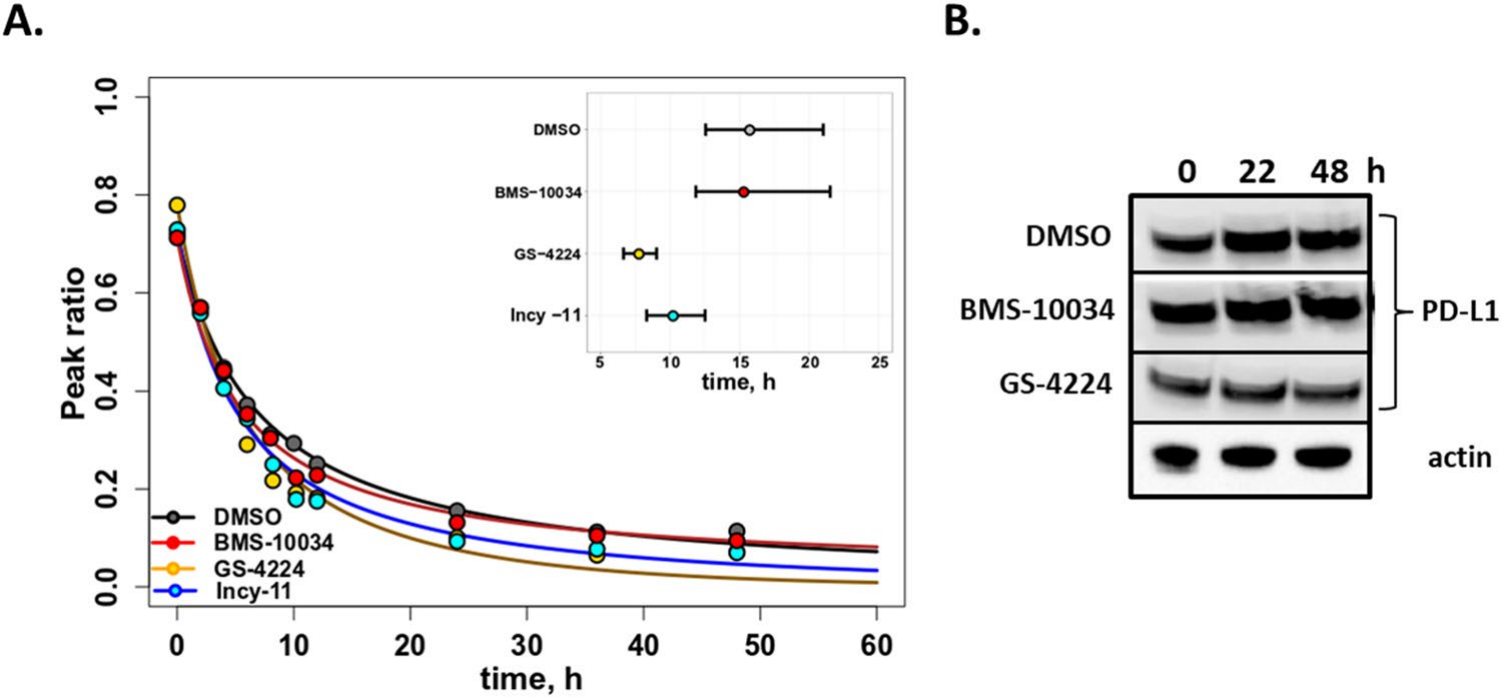
Overall turnover of PD-L1 protein. (**A**) Degradation of PD-L1 3xFLAG protein transfected into HEK 293 T cells measured by SILAC pulse chase method. Data for DMSO control are superimposed with those for dimer-inducing 1 μM GS-4224 and Incy-11, and 1 μM macrocyclic blocker BMS-10034. The half-life comparisons demonstrate that DMSO and BMS-10034 have half-life estimates that are not statistically different (p > 0.10) yet these are statistically greater (p < 0.0001) than the GS-4224 and Incy-11 half-life estimates. Also, GS-4224 has a half-life estimate that is statistically less (p < 0.001) than that of Incy-11. (**B**) Total endogenous PD-L1 levels in MDA-MB-321 GFP cells measured over 48 h by WB. GS-4224 treated samples are compared to DMSO control and BMS-10034 treated cells. Actin is used as a loading control.

Transiently transfected human embryonic kidney (HEK) 293 T cells were pulsed for 12 h with heavy amino acids (^13^C_6_ l-Arginine and ^13^C_6_ l-Lysine) and were treated during the chase with PD-L1 dimer-inducing compounds (GS-4224, INCY-11), a non-dimerizing inhibitor (BMS-10034), or a vehicle control. The ratio of heavy to light peptide peak area determined for 7 PD-L1 peptides was used to calculate an average labelling of PD-L1. After cell-doubling correction, the half-life of PD-L1 was approximated to be 15 h in non-dimerizing versus 10 h under dimer-inducing conditions (Fig. 2A). These results were reproduced in three separate biological experiments, and statistical significance of the measurements was verified using the Mann–Whitney-Wilcoxon test in R. This small yet consistent change in the relative degradation rate of the PD-L1 prompted additional investigations of the PD-L1 cellular distribution.

To further evaluate the total levels of PD-L1 protein under various treatment conditions, western blot (WB) detection for a human breast cancer cell line expressing high level of PD-L1 endogenously (MDA-MB-231 GFP) was used. Total PD-L1 levels were assessed at 24 h and 48 h post treatment under the following conditions: DMSO control, a dimer inducer (GS-4224), and a macrocyclic blocker (BMS-10034). No statistically significant change in the amount of detected protein was observed for any of the assessed conditions (Fig. 2B). These data are consistent with the PD-L1 degradation rates obtained for the transiently transfected system, as the small changes observed in SILAC pulse chase measurements, are outside the precision range for the WB technique. We next examined whether a change in cellular distribution of PD-L1 on plasma membrane versus cytoplasm could explain the difference in degradation kinetics.

### PD-L1 cellular localization

**Figure 3:**
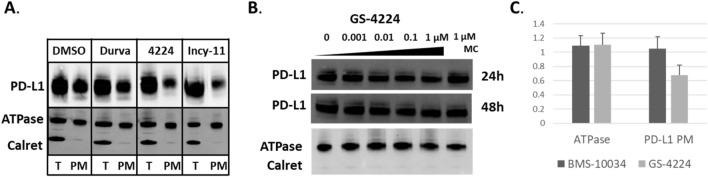
WB analysis of reduction in PD-L1 on the cell surface. (**A**) PD-L1 levels in plasma membrane isolated from MDA-MB231 GFP. WB signal from samples treated with durvalumab, GS-4224, or INCY-11 is compared to the one obtained for the DMSO control. Na^+^/K^+^ ATPase is used as the plasma membrane marker, and calreticulin as the ER marker. T stands for total protein fraction, PM stands for plasma membrane fraction. (**B**) Titration of GS-4224 compound in MDA-MB-231 GFP cells. Tenfold serial dilution was used with a concentration range: 1 nM–1 µM and with samples collected at 24 h and 48 h. Annotation is as in (**A**), and MC stands for macrocycle, BMS-10034. (**C**) Bar graph comparison of WB signal intensity for PD-L1 plasma membrane protein in MDA-MB-231 GFP cells treated with either 1 µM GS-4224 or 1 µM BMS-10034. Na^+^/K^+^ ATPase is used as a control for general plasma membrane protein levels. Cropped and grouped blots here and in all the following figures are explicitly delineated either with dividing lines or white space. All uncropped blots are included in the Supplemental Information.

To assess plasma membrane proteins by WB, all proteins are first biotinylated on the surface of intact cells, followed by cell lysis and enrichment of plasma membrane proteins via a streptavidin pull-down step. Different modes of PD-L1 blockade were again examined: a blocking antibody (Durvalumab analogue) and dimer inducing compounds GS-4224 and INCY-11. After treatment, plasma membrane proteins, including PD-L1, were enriched and the amount of isolated PD-L1 protein was quantified by WB. Na^+^/K^+^ ATPase, an unrelated plasma membrane protein, was used as a positive control for enrichment, while the endoplasmic reticulum (ER)-localized calreticulin, was used as a negative control to ensure that only plasma membrane proteins were indeed enriched upon biotinylation. As expected, constant levels of Na^+^/K^+^ ATPase were observed after plasma membrane enrichment, while those of calreticulin were depleted. Treatment with dimer-inducing inhibitors showed approximately 40% reduction in PD-L1 associated with the plasma membrane compared with DMSO and antibody controls (Fig. 3A).

To further confirm the magnitude of change in PD-L1 content of the plasma membrane fraction, GS-4224 compound was applied at concentrations ranging between 1 nM and 1 µM, and protein levels were assessed after 24 h of incubation. In a separate experiment the titration treatment was also extended to 48 h (Fig. 3B). For these experiments, the macrocycle (MC) BMS-10034 was used as a non-dimer inducing control. For all examined concentrations at both time points, the data are consistent with a less than a two-fold reduction in PD-L1 plasma membrane levels (Fig. 3C). The observation of similar effects at all tested GS-4224 concentrations is consistent with the GS-4224 dimerization EC50 for PD-L1 protein in MDA-MB231 measured at 1.4 nM ± 0.5 (*manuscript in preparation*), and no enhancement in protein depletion takes place at higher concentrations. Between the two experiments (Fig. 3A,B), the change in the PD-L1 protein levels was consistent with a range of 20–40% overall reduction. Due to limitations associated with interpreting WB quantification when dealing with changes that are less than twofold, no further quantitative analysis was performed. However the observation that PD-L1 protein is indeed being mildly depleted from the surface while the overall levels remain unchanged suggests that the protein may to some extent accumulate intracellularly, and an orthogonal method was used for further investigation.

**Figure 4:**
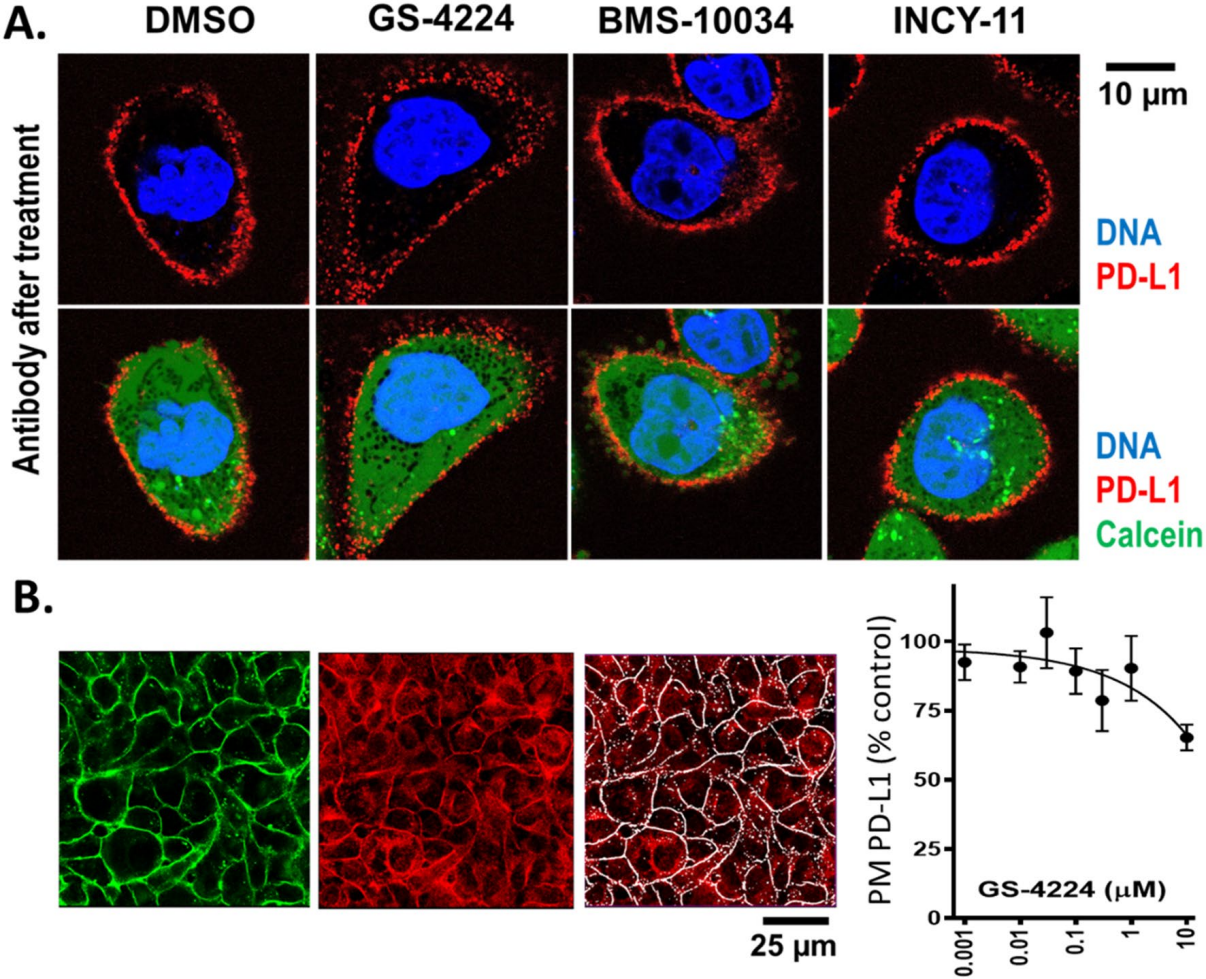
Microscopy analysis of PD-L1 cellular localization. (**A**) CHO cells overexpressing PD-L1, which is detected with anti-PD-L1 antibody (red). Intracellular space and nuclei stained with Calcein AM (green) and Hoechst (blue), respectively. The detection antibody was applied after 1-h of compound treatment. (**B**) MDA-MB-231 cells treated with GS-4224 for 24 h, fixed and stained with anti-PD-L1 antibody (red). Cell surface mask (white) was created using an anti-Na^+^/K^+^-ATPase antibody staining (green). PD-L1-specific signal at cell surface is plotted against concentration of dimerizing compound GS-4224.

Cellular distribution of PD-L1 protein upon dimerization was assessed by confocal microscopy for two cell lines: CHO, a cell line stably expressing recombinant PD-L1 protein, and MDA-MB-231 with endogenous PD-L1 protein. PD-L1 was detected using immunofluorescence in cells treated with either BMS-10034, a compound known to bind to PD-L1 with 1:1 stoichiometry, or with one of two distinct chemical scaffolds known to bind PD-L1 dimers in a 1:2 stoichiometry (GS-4224 and INCY-11). Final concentration of inhibitors was 1 µM. Figure 4A shows that, regardless of the compound applied, PD-L1 was still present on the surface of CHO cells expressing full-length human PD-L1 after 60 min of treatment at 37 °C. PD-L1 on the surface of live cells was labelled with anti-PD-L1 antibody directly conjugated to fluorescent dye Alexa Fluor 647 and the intracellular space was stained with Calcein AM. No substantial rapid internalization of PD-L1 occurred upon dimerization of the protein, and a strong PD-L1 signal was still detected on the surface of the CHO-PD-L1 cells treated with either GS-4224 or INCY-11 for 6 h or 24 h, although to lesser extent than in cells treated with DMSO (Suppl. Figs. S1 and S2).

To exclude possible artefacts associated with an overexpression system, MDA-MB-231 cells were analysed for localization of endogenous PD-L1. The cells were sucted to a 24-h treatment with the dimerizing compound GS-4224, fixed in paraformaldehyde, and stained with the anti-PD-L1 antibody. Antibody against Na^+^/K^+^ ATP-ase was used as a plasma membrane marker for subsequent image analysis. To ensure that the observed fluorescence reflects specific binding of the anti-PD-L1 antibody to the target protein, the MDA-MB-231 GFP PD-L1 knock-out (KO) cell line was used as a negative control (Suppl. Fig. S3). As shown in Fig. 4B, the levels of PD-L1 protein in the plasma membrane were minimally affected after 24 h of treatment except at the highest inhibitor concentration (10 µM), where a ~ 35% reduction in the PD-L1-specific cell surface signal was observed. Thus, in this set of experiments as well, the majority of PD-L1 remains on the plasma membrane despite the prolonged treatment with a dimerizing compound.

### Experiment-specific internalization

**Figure 5:**
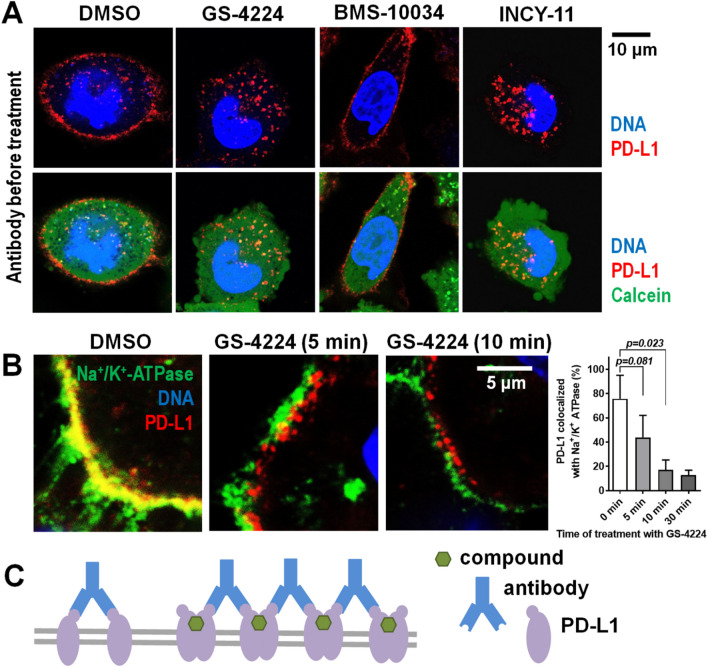
Internalization of PD-L1 induced by combination of dimerizing compounds and non-neutralizing antibody. (**A**) PD-L1-expressing CHO cells were pre-incubated with fluorescently-labeled (red) anti-PD-L1 antibody for 20 min following 1-h treatment with compounds at 37 °C and then imaging at 21 °C. The intracellular space was stained with Calcein AM to visualize cell boundaries. (**B**) High-magnification confocal images of MDA-MB-231 cells demonstrating the fast loss of colocalization of Na^+^/K^+^-ATPase (plasma membrane marker, green) and PD-L1 (detected with fluorescently labeled antibody, red) when antibody was applied prior to the dimer-inducing compound GS-4224. The graph shows the percentage of PD-L1 co-localized with Na^+^/K^+^-ATPase at different time of incubation with GS-4224. Data are mean ± S.D. (n = 3–5). (**C**) A schematic of the postulated crosslinking which may explain experiment-specific internalization of dimerized PD-L1.

The confocal microscopy data described above were obtained when the detection antibody was applied shortly prior to the imaging but after completion of incubation with PD-L1 inhibitors and cellular fixation. Interestingly, images consistent with rapid PD-L1 internalization were obtained for the cells pre-incubated with anti-PD-L1 antibody before the application of the dimer-inducing molecules (Fig. 5).

For instance, in MDA-MB-231 cells fixed at various time points during the treatment with GS-4224 in the presence of anti-PD-L1 antibody, the PD-L1-positive vesicles promptly appeared in the intracellular space near the plasma membrane (Fig. 5B). The analysis of high magnification images of the regions near the cell edges demonstrated that the portion of PD-L1 colocalized with Na^+^/K^+^-ATPase declined sharply from 75.7 ± 19.4% in DMSO-treated samples to 43.9 ± 18.1%, 17.2 ± 8.1% and 12.7 ± 4.1% after 5, 10, and 30 min incubation with the dimerizing compound, respectively (mean ± S.D., n = 3–5 cells).

We also assessed the distribution of PD-L1 by analyzing images containing several cells (Suppl. Fig. S4). The percentage of PD-L1 colocalized with plasma membrane marker Na^+^/K^+^ ATPase was lower in this case but the inhibitor-induced decrease in the value of the parameter was significant (37 ± 14.6% and 17.3 ± 6.3% PD-L1 was colocalized with Na^+^/K^+^-ATPase in DMSO-treated and compound-treated (1 µM, 30 min) cells, respectively (n = 7–12 microscopic fields, *p* = 0.011). The data suggest that the presence of non-neutralizing anti-PD-L1 antibody caused the internalization of antibody-PD-L1 complex upon addition of a dimerizing agent.

The live cell imaging performed for CHO cell line expressing human full-length PD-L1 treated with the inhibitors after pre-incubation with anti-PD-L1 antibody (Fig. 5A) demonstrated disappearance of PD-L1 from the cell surface in the presence of the dimerizing compounds (GS-4224 and INCY-11) but not in the presence of the macrocycle compound (BMS-10034), contrary to the result obtained when the antibody was applied after treatment with the compounds (Fig. 4A). This procedure-specific observation can be attributed to cross-linking induced internalization and is further discussed below.

### Intracellular retention of the PD-L1 dimer

**Figure 6:**
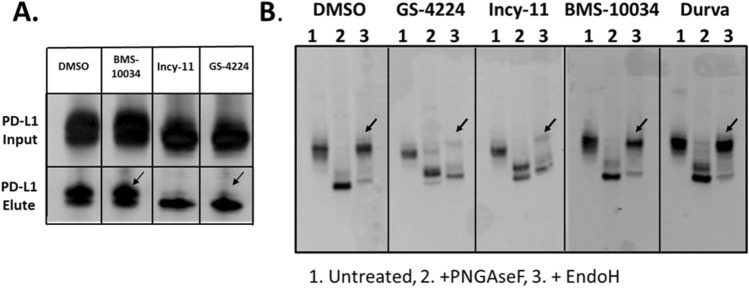
PD-L1 glycosylation differences associated with compound treatments. (**A**) WB signal for FLAG-PD-L1 in HEK293 T cells overexpression system. A high molecular weight glycosylation species is absent from PD-L1 samples treated with dimer-inducing compounds (arrows). (**B**) Dimerized PD-L1 is more susceptible to EndoH than monomeric PD-L1 (arrows indicate the EndoH-resistant species).

Having ruled out physiological internalization, we next looked at post-translational modifications known to have a role in the PD-L1 lifecycle. In our IP experiments with overexpressed FLAG-tagged PD-L1 protein, we noted that two distinct species of PD-L1 were resolved, with the higher molecular weight species diminishing under dimerization conditions only (Fig. 6A). We confirmed that both species are indeed PD-L1 by performing a set of experiments aimed at removal of protein glycosylation. Upon PNGaseF treatment, which does not differentiate between glycan composition, a single molecular weight band was observed indicating that differential glycosylation pattern was responsible for the apparent difference in molecular weight (Fig. 6B). However, the results of the digestion with EndoH, which is active on high-mannose sugar moieties only, revealed that there is a difference between samples depending on the treatment. In this case, samples treated with dimerizing compounds demonstrated higher susceptibility to EndoH, suggesting that a higher proportion of the protein resided in the ER rather than on the plasma membrane.

**Figure 7:**
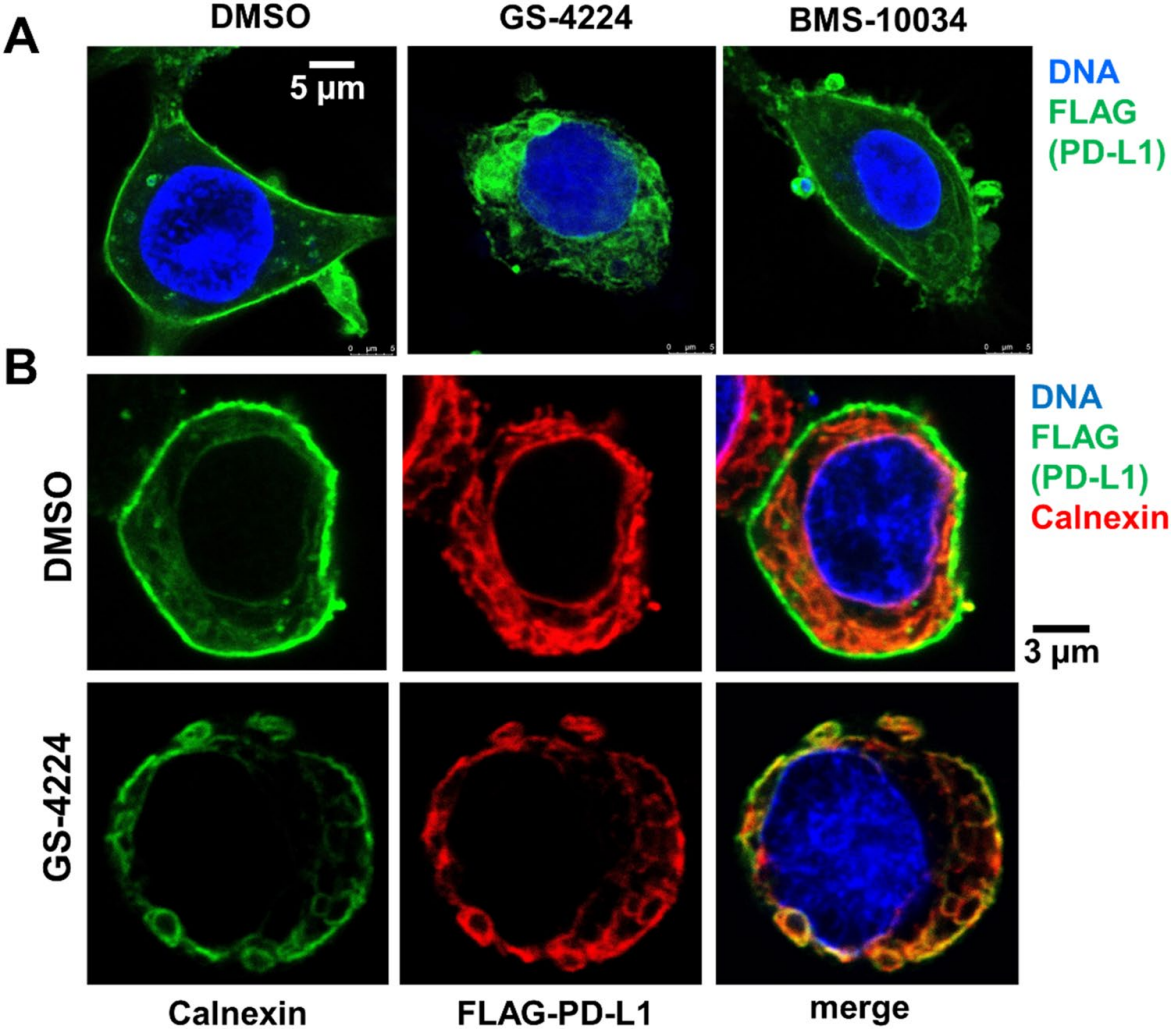
Intracellular accumulation of PD-L1. (**A**) Expi293 cells overexpressing FLAG-PD-L1, which is detected with anti-FLAG antibody (green). Nuclei are stained with Hoechst (blue). (**B**) Expi293 cells treated with GS-4224 for 24 h, fixed and stained with anti-FLAG antibody (green) to detect PD-L1; anti-calnexin antibody (red) to label ER, and Hoechst (blue) to visualize nuclei.

The potential retention of newly synthesized PD-L1 protein in cytoplasm was confirmed for Expi293 cells transiently expressing human FLAG-PD-L1 by utilizing confocal microscopy. We rationalized that an overexpression system would amplify defects in ER to PM trafficking allowing for a better differentiation of the relative impact of the different PD-L1 binders. The initial experiments revealed that, treatment with the dimerizing compound GS-4224 but not the macrocycle BS-10034 led to reduction of FLAG-PD-L1 at the cell surface and to a significant accumulation of the protein in intracellular structures resembling ER (Fig. 7A). Thus, we performed a colocalization experiment using calnexin as an ER marker.

Following a 24 h period after transfection, the cells were incubated with either DMSO or GS-4224 for another 24 h, fixed and stained to visualize both FLAG and calnexin. DMSO-treated cells had strong FLAG-specific fluorescence signal at their surface. The anti-FLAG antibody also detected intracellular FLAG signal, which was colocalized with the ER marker and was likely associated with recently produced recombinant FLAG-tagged PD-L1. The average percentage of FLAG-PD-L1 colocalized with calnexin was 41.2 ± 22.1% and 77.9 ± 21.4% for cells treated with DMSO or 100 nM GS-4224, respectively (mean ± S.D., n = 21–37 cells, *p* < 0.001). As shown in Fig. 7B, FLAG-PD-L1 is depleted at the surface of the cells treated with GS-4224 and is detected in calnexin-positive (ER) regions. We also observed modest cytoplasmic PD-L1 accumulation in MDA-MB-231 GFP cells treated with 10 µM GS-4224 for 24 h (d) Suppl. Fig. S5). Taken together these data strongly suggest that compound-induced dimerization impairs PD-L1 trafficking to the plasma membrane.

## Discussion

PD-1/PD-L1 checkpoint blockade for oncology indications has been a success story of the last decade with seven antibody treatments approved for clinical use and many more in the pipeline^1,39,40^. Along with indisputable benefits of this treatment, opportunities for improvement also became apparent. Two notable issues are resistance or low objective response rate in certain patient populations and the general cost associated with antibody therapies.^7,41,42^.

Thus, there has been a continuous effort to develop small molecule blockers of the PD-1/PD-L1 interaction as these treatment options could offer advantages such as oral availability, combination potential and lower cost of production, as well as potentially novel mechanistic approaches^12,43–46^. One class of small molecule PD-L1 inhibitors has been shown to induce PD-L1 dimerization, and there are currently two such dimer-inducing compounds in clinical trials^27,47^. Unlike blocking therapies, this novel mechanism functions by disrupting PD-1/PD-L1 interaction through dimer-induced PD-1 binding site occlusion.

Interestingly, while there has been a great deal of activities associated with PD-1/PD-L1 blockade for clinical development, our understanding of these receptors at the molecular level remains limited. Therefore, general effects of PD-L1 dimerization on PD-L1 trafficking or turnover are difficult to predict. PD-L1 is known to function as a monomer on the cell surface, and only recently has been reported to interact with other proteins on the same cell surface (in cis configuration). For example, recent studies have shown that PD-L1 forms cis-heterodimers with CD80^48,49^ as well as specific interactions with other membrane associated proteins such as chemokine-like factor-like MARVEL transmembrane domain-containing 4/6 (CMTM4/6)^34,50^. PD-L1 cellular stability has been shown to be linked to CMTM protein interactions, as well as to post-translational modifications of the PD-L1 protein as comprehensively reviewed by Yu et al. Biomedicines 2021^51–54^. A dimerization event was postulated to influence PD-L1 protein interactions and stability in a recent study involving the natural product, resveratrol^55^, and testing such observations for synthetic inhibitors known to induce dimerization provided further motivation to examine inhibitor-induced PD-L1 dimer cellular lifecycle.

In this work we describe the effects of PD-L1 oligomerization on turn-over kinetics, cellular distribution, and glycosylation. To draw more general conclusions about the protein lifecycle, the described experiments span different expression systems, and include analysis of transiently expressed PD-L1 in HEK293T cells, stably expressed PD-L1 in CHO cells, and finally endogenous protein in MD-MBA-231 GFP cancer cell line. We include observations of experiment-specific phenomena that may not reproduce in orthogonal studies^25,26^, thereby emphasizing the need for caution and extensive experimentation when working with newly emerging systems.

Our analysis of PD-L1 dimer life cycle indicates that the overall degradation rate of this protein is increased by 30% as compared to its monomeric counterpart in a transiently transfected system. This subtle, but consistent change in turn-over for the long-lived PD-L1 protein, is not resolved by western blot when examining endogenous protein in MDA-MB-231 GFP, and as expected, the total PD-L1 levels remain similar between oligomeric states 48 h post dimerization (Fig. 2). However, the subtle change in protein degradation translates into more pronounced effects when examining the compartmentalization of PD-L1. Interestingly, a consistent decrease in plasma membrane protein levels is observed across all cellular systems tested and with supporting data generated by two orthogonal methods, western blot (Fig. 3) and microscopy analyses (Fig. 4). This observation suggests that while the overall kinetics may be minimally affected, the protein is redistributed due to the dimerization event. Decrease in plasma membrane protein would then suggest retention intracellularly, and this latter point is corroborated by the examination of glycosylation profiles for the various samples (Fig. 6). Indeed, cells treated with PD-L1 blockers show resistance to EndoH treatment, indicating that the protein has moved past the ER compartment and most likely onto the cell membrane. This observation is starkly different from cells treated with dimer-inducing compounds, where most of the PD-L1 is susceptible to EndoH treatment and points to ER retention. Hence, for the first time, we can conclude that the effects of dimer-inducing inhibitors are rendered not only on the cell membrane but also intracellularly, with the intracellular dimer formation resulting in ER retention. These lifecycle changes are unique to dimer inducing compounds and are not observed for either small or large molecule blockers, BMS-10034 and Durvalumab analogue, respectively. The retention is corroborated by microscopy data (Fig. 7), which also suggest that the extent of intracellular retention is consistent with the levels of expression of PD-L1. MDA-MB-231 GFP cells, which express lower levels of PD-L1, show less retention than higher expressing stably transfected CHO cells. The observations of the change in the PD-L1 lifecycle align well with the phenotype reported by Verdura et al. where the authors hypothesize a dimer-induced retention mechanism at play for a natural product, resveratrol^55^.

We also detect internalization as recently reported by several groups^24–26^. However this phenomenon is observed only under a very specific set of experimental conditions. We explain the internalization of dimerized PD-L1 by the antibody-mediated crosslinking, consistent with experimental set up described by Park et al.^25^ and Koblish et al.^26^ where detecting antibodies were applied prior to fixation (Fig. 5). Applied separately, small molecule and non-neutralizing antibody can only produce dimeric PD-L1 complexes while combination of these two agents creates a situation when the presence of antigens with two epitopes (PD-L1 dimers) leads to crosslinking and formation of large PD-L1 clusters, which may internalize via endocytosis resulting in removal of PD-L1 from the cell surface. It may indicate a potential for use of such a combination (dimerizing small molecule and non-blocking anti-PD-L1 antibody) as a strategy to boost removal of PD-L1 from the cell surface. Despite the obvious appeal of such a removal from therapeutic standpoint, our studies indicate that the dimerizing compounds applied alone (prior to the detection antibodies) do not cause rapid internalization of neither endogenous nor transfected PD-L1. Long-term effects of PD-L1 dimerization also did not manifest as disappearance of PD-L1 from the plasma membrane, as the protein was still detectable in PD-L1-positive MDA-MB-231 GFP cells after 24 h and 48 h of exposure to GS-4224. Similar to the observations reported by Snapp et al.^54^, using EYFP-fused PD-L1^56^ resulted in formation of ER whorls or Organized smooth ER (OSER) in our experiments with dimerizing compounds (Suppl. Fig. S6), a known consideration to be aware of when working with fusion proteins with tendency to dimerize^57,58^. Hence the observation of antibody-induced internalization in literature should be interpreted with caution as interaction of reagents and compounds with novel mechanisms of action could resulted in unexpected behavior.

Overall, our data suggest that dimerization of PD-L1 does not considerably change the life cycle of PD-L1 protein in the short term (< 48 h). While we see a small enhancement in PD-L1 degradation and a corresponding reduction in PD-L1 levels on plasma membrane, this change is not due to internalization of the receptor and is instead consistent with ER retention. The latter observation points to the fact that dimer-inducing compounds modulate not only on the protein already established on the cell membrane, but also the new synthesized PD-L1 associated with the intracellular compartments. This evidence serves to expand the function of the dimer inducing class of small molecule inhibitors beyond plasma membrane, potentially opening avenues for future exploration.

## Methods

The syntheses of compounds used in this study are described in the patent literature^13–19^. The compounds’ naming corresponds to the location of their description in respective patent application: BMS-10034 from Bristol Myers Squibb, INCY-11 from Incyte Corporation, and GS-4224 from Gilead Sciences Inc.The PD-L1 blocking antibody, (Durvalubamab analogue) was produced in-house. The heavy and light chain sequences of the antibody were downloaded from Drugbank. DNA encoding heavy chain and light chain sequences was synthesized by GeneArt and cloned into pcDNA3.1 expression vector. Expression of the Durvalumab analogue, was performed in Expi293F cells (Thermo Fisher Scientific) according to manufacturer’s protocol, and purification was performed following standard protocols.

### PD-L1 degradation measurement through Stable Isotope Labeling with Amino acids in Cell culture (SILAC)

*SILAC pulse chase treatment.* HEK293T cells (ATCC CRL3216) were adapted to SILAC R0K0 media (SILAC Dulbecco’s Modified Eagle Medium (DMEM) from ThermoFisher Scientific A33822, 10% Dialyzed FBS, 1% Pen/Strep, 1% l-Glut, 0.398 mM l-Arginine, 0.798 mM l-Lysine Hydrochloride) by passaging a minimum of five times in media and then seeded in 6 well plates for each treatment where each well represented one timepoint. A total of 3 × 10^5^ cells was plated into each well. Cells were transfected with 1.67 µg of pFUSE full length human PD-L1 3X FLAG 24 h post seeding. SILAC R0K0 media was then replaced at 10–12 h post transfection with SILAC R6K6 media (SILAC DMEM, 10% Dialyzed FBS, 1% Pen/Strep, 1% l-Glut, 0.398 mM ^13^C_6_ l-Arginine, 0.798 mM ^13^C_6_ l-Lysine Hydrochloride) for the pulse. Media was then changed back to SILAC R0K0 media at 24 h post transfection. Treatment with PD-L1 inhibitors was implemented concurrently with the chase. All inhibitor treatments were added to a final concentration of 1 µM, and timepoints were taken thereafter over the course of 12 or 48 h (Suppl. Fig. S7). Cells were harvested by resuspension in 1 mL of PBS and were centrifuged at 524×*g* for 5 min at room temperature. Cells were counted in triplicate using the Thermo Fisher Applied Biosystems Countess II to monitor the cellular growth rate. Media and PBS were aspirated off, and the cells were washed again with 1 mL of PBS and centrifuged in 1.5 mL Eppendorf tubes at 0.2 g for 5 min at 4 °C. All samples were stored at − 80 °C until the conclusion of the time course.

#### Pulse chase sample processing

Cells were thawed on ice and resuspended in 200 µL of Lysis buffer (50 mM HEPES pH 7.5, 150 mM NaCl, 0.1% SDS, 1% NP-40 supplemented with 1 complete mini EDTA free protease inhibitor [Roche] tablet per 5 mL of buffer). Samples were lysed via a 27.5-gauge syringe, incubated on ice for 10 min, and then centrifuged at 21.1×*g* for 10 min at 4 °C to pellet cellular debris. The supernatant for each timepoint was then loaded onto 30 µL of magnetic M2 FLAG beads (Sigma) preequilibrated with PBS buffer and incubated overnight with rocking at 4 °C. The next day, unbound sample was collected, and the beads were washed three times with 1X PBS and centrifuged at 0.2 g for 5 min at 4 °C between each wash. To elute, the beads were resuspended in 60 µL (2CV) of 0.1% formic acid in water and incubated at room temperature (RT) for one hour.

#### Sample processing for mass spectrometry (MS) analysis

20 µL of eluate from each timepoint was transferred to a 96 well 200 µL Axygen plate. Samples were neutralized with the addition of 2 µL of 1 M Tris pH 8.0. 3 µL of 0.1% ProteaseMax (Promega, Madison, WI) was added to enhance subsequent trypsin digestion and incubated at 95 °C for 2 min. Disulfide bonds in the samples were then reduced with the addition of 200 mM DTT to a final concentration of 4 mM and incubated at 60 °C for 30 min. Cysteines were alkylated with 1 µL of 400 mM Iodoacetamide and incubated in the dark for 30 min at room temperature. 1 µL of 0.5 mg/mL Trypsin (Promega) was added to each sample and the samples incubated overnight at 37 °C. The trypsin digestion was stopped via addition of 2 µL of 1% trifluoroacetic acid (TFA). 2 µL of each sample was then analyzed by mass spectrometry.

#### LCMS data acquisition and analysis

The HPLC used for peptide separation was a Dionex UltiMate 3000 UHPLC (ThermoFisher Scientific. Waltham, MA). The analytical column was an EasySpray 75 µm × 25 cm PepMap RSLC 2 C18 µm column (ThermoFisher Scientific) and the trapping column was a 0.3 mm × 5 mm PepMap 100 C18 column (ThermoFisher Scientific). The peptide sample was loaded onto the trapping column with an isocratic gradient of 1% acetonitrile, 0.1% TFA for 5 min at 20 µL/min. The trapping column was then switched in-line with the analytical pump and column. The analytical gradient was from 2 to 32% of Buffer B (0.1% formic acid in acetonitrile) in 21 min, followed by a gradient from 32 to 80% Buffer B in 2 min, a wash step of 80% Buffer B for 2 min, and finally a re-equilibration step of 2% Buffer B for 15 min. Buffer A consisted of 0.1% formic acid in water. Peptides were ionized by electrospray into the mass spectrometer.

The mass spectrometer was an Orbitrap Fusion Lumos (Thermo Fisher Scientific). Data was acquired with a 1.5 s scan cycle of a MS1 full scan followed by data-dependent MS2 scans. The MS1 scan was acquired at 120,000 resolving power from 350 to 2000 m/z, an AGC of 4e5 ions and a maximum injection time of 50 ms. MS2 scans were acquired with HCD fragmentation, 27% CE, and a resolving power of 15,000.

Peptides were identified from a database search of MS2 mass spectra using the Sequest HT algorithm (Proteome Discoverer 2.4, Thermo Scientific) against the PDL1-FLAG sequence concatenated with sequences of common protein contaminants, with a precursor mass tolerance of 10 ppm and a fragment mass tolerance of 0.02 Da. Peak areas from all PDL1-FLAG peptides identified from the database search were used for turnover calculations [AALQITDVK (2 +), NIIQFVHGEEDLK (2 + , 3 +), DQLSLGNAALQITDVK (2 + , 3 +), LQDAGVYR (2 +), ILVVDPVTSEHELTC[+ 57]QAEGYPK (3 +), AEVIWTSSDHQVLSGK (3 +), C[+ 57]MISYGGADYK(2 +), VNAPYNK (2 +), QLDLAALIVYWEMEDK(2 + , 3 +), C[+ 57]GIQDTNSK (2 +), QSDTHLEETGGGGSDYK(2 +)]. Precursor peak areas (M, M + 1 and M + 2) were extracted using Skyline^59^.

For each sample, peptide peak areas were filtered out if the summed total of the unlabeled and labeled precursor peak area was less than 5E5. The overall labeling ratio for PD-L1 for each time point was the average of heavy to total peptide area for each PD-L1 peptide detected. Labeling ratios for each treatment were then fitted to a non-linear differential equation in R. Cell growth rates were estimated by fitting a logistic growth model, $\mathrm{dN}/\mathrm{dt}=\mathrm{rN}\left(1-N/K\right)$ where $\mathrm{r}$ is the cell ($\mathrm{N}$ is number of cells) growth rate in media and $\mathrm{K}$ is the media carrying capacity (maximum cell population capacity in untreated media) to the cell counts obtained over the course of the experiment. The growth rates were then subtracted from the labeling rates to obtain the protein degradation rates. The subtraction of the growth rate was necessary to better approximate the degradation rate of PD-L1 with and without treatment because the cells doubled multiple times over the course of the experiment. Data were plotted and analyzed using R. The equation $dN/dt=r\left(1-N/cK\right)-\alpha N$*,* was used, where α is the rate of decay, $c$ is how the treatment alters K and therefore the half-life is given by ln(2)/α.

### Western blot analysis of PD-L1 Levels

To assess total PD-L1 levels as well as those in the plasma membrane fraction upon treatment with small molecule modulators, MDA-MB321 GFP cell line obtained from Creative Biogene (Shirley, New York) producing high endogenous levels of this protein was used. For total PD-L1 protein, 6-well plates were seeded with 10 × 10^5^ MDA-MB-231 GFP cells, grown for 12 h and treated with 1 µM compound (GS-4224 or BMS-10034). Cells were harvested and analysed by standard Western Blot (WB) protocols post 24 h or 48 h of treatment. Antibodies used for this analysis were purchased from Abcam: β-actin (ab8224) and PD-L1 (ab213524), with β-actin used as a loading control. Thermo Scientific™ Pierce™ ECL 2 Western Blotting Substrate kit was used for detection with anti-mouse or anti-rabbit HRP conjugated secondary antibodies.

Pierce Cell Surface Protein Isolation Kit (Thermo Scientific) was used to isolate plasma membranes per manufacturer’s instructions. For these experiments, 0.5 × 10^6^ MDA-MB-231 GFP cells were seeded in a T75 cm^2^ flask in DMEM media and incubated at 37 °C for 3 days. For initial experiments, after the culture became ~ 80% confluent, the flasks were treated with either 1 µM GS-4224, 1 µM INCY-11, 10 µg/mL durvalumab analogue or DMSO and further incubated for 24 h. Two T75 cm^2^ flasks were used per each condition. After the incubation period, the cell surface was biotinylated and surface proteins isolated following the exact protocol described for the kit. For the follow-up experiments the same growth conditions were used, but the cells were treated with a tenfold serial dilution of GS-4224 concentrations, ranging from 1 nM to 1 µM, for either 24 h or 48 h. BMS-10034 (1 µM) and DMSO (1%) were used as non-dimer inducing controls.

Isolated fragments of plasma membrane were subjected to WB analysis. Antibodies used for this analysis were purchased from Abcam: β-actin (ab8224), Na^+^/K^+^ ATPase (ab76020), calreticulin (ab92516), PD-L1 (ab213524). Specificity of PD-L1 antibody was further verified using MDA-MB-231 GFP PD-L1 KO cell line generated using CRISPR technology (B-MoGen Biotechnologies). Actin was used to assess total protein loading, while Na^+^/K^+^ ATPase and calreticulin were used as plasma membrane and ER localization markers, respectively. Thermo Scientific™ Pierce™ ECL 2 Western Blotting Substrate kit was used for detection with anti-mouse or anti-rabbit horseradish peroxidase-conjugated secondary antibodies.

### PD-L1 localization by confocal microscopy

MDA-MB-231 GFP or Expi293 cells were seeded onto glass coverslips (Corning BioCoat Poly-d-Lysine/Laminin 12 mm, Corning, NY) placed in 12-well Corning plates. Cells were fixed in ice-cold methanol for at least 15 min and washed three times in Dulbecco's Phosphate-Buffered Salt Solution (DPBS) (Corning). The immunostaining was performed at RT. Bovine serum albumin (2% BSA, MilliporeSigma) was used for blocking. Primary and secondary antibodies were then applied to cells for 90 min and 60 min, respectively. The following primary antibodies were used: anti-PD-L1 antibody (Abcam, ab209960, clone 28-8 conjugated with Alexa Fluor 647, 2.5 μg/mL), Na^+^/K^+^ ATPase antibody (ab7671, 2.5 μg/mL), anti-FLAG M2 antibody conjugated to cyanine dye Cy3 (MilliporeSigma 9594, 6.7 μg/mL), anti-calnexin antibody (ThermoFisher PA5-34754, 5 μg/mL). Secondary antibodies conjugated with either Alexa Fluor 488, Alexa Fluor 633 or Alexa Fluor 647 were purchased from ThermoFisher Scientific and used at 2 mg/mL after dilution in DPBS containing 1% BSA. The coverslips with stained cells were mounted on the glass microscopic slides (VWR International, Radnor, PA) with a drop of ProLong Gold antifade reagent containing DAPI (ThermoFisher Scientific).

The samples were imaged with confocal laser scanning microscope Leica SP8 (Leica Microsystems Inc., Wetzlar, Germany) equipped with a white laser for excitation and HyD detectors. Images within each sample set were captured using identical instrument settings. The acquisition was performed using several sequences to minimize bleed-through artefacts. To remove operator bias, an approach analogous to the quadrat sampling was applied in FLAG/Calnexin colocalization studies. The tiling (3 × 3) allowed for registration of the positions of the cells outside of the central microscopic field and thus invisible during focus adjustment. Then these cells, selected in unbiased way, were imaged individually with high resolution.

The experiments aiming at assessing the compound-induced internalization of PD-L1 were conducted with CHO cells (Promega) stably expressing recombinant PD-L1 and cultured in 96-well plates with the coverslip-glass bottom (Eppendorf, Cat#0030741030). Although incubation with the inhibitors was conducted at 37 °C, the consecutive live cell imaging was performed at 22 °C to slow down the endocytosis and minimize the artefacts associated with the antibody-induced internalization of PD-L1 dimers. Anti-PD-L1 antibody conjugated with Alexa Fluor 647 (Abcam, ab209960) was used to detect PD-L1. The intracellular space and nuclei were labeled using Calcein AM (ThermoFisher Scientific) and Hoechst 3342 (ThermoFisher Scientific), respectively.

The images were analyzed with either Image J (NIH, Bethesda, MD) or Imaris (Oxford Instruments, Abingdon, United Kingdom) software. The amount of colocalized “material” was assessed by accounting for the number of colocalized pixels and their intensity. Images were adjusted equally within each data set for brightness, contrast and sharpness using Leica Application Suite X (LAS X) and PowerPoint 2010 (Microsoft, Redmond, WA, USA).

### Glycosylation analysis of PD-L1

The HEK293T cells transfected with FLAG-tagged PD-L1 as described above for the SILAC degradation experiments were also used to assess PD-L1 glycosylation levels. Cell pellets were prepared as described above, and lysates and IP elution fractions subjected to PNGaseF (New England Biolabs, P0710S) or EndoH (New England Biolabs, P0702L) treatment per manufacturer’s instructions.

## Supplementary Information

Supplementary Information.

## Data Availability

All data generated or analysed during this study are included in this published article [and its supplementary information files].
