# Supplementary material for: Effects of small molecule-induced dimerization on the programmed death ligand 1 protein life cycle
Source: Sci Rep. 2022 Dec 9;12:21286. doi: 10.1038/s41598-022-25417-6 (PMC9734112; doi:10.1038/s41598-022-25417-6)
Supplement: Supplementary file 1 — Supplementary Information. [file 41598_2022_25417_MOESM1_ESM.pptx]

## Slide 1
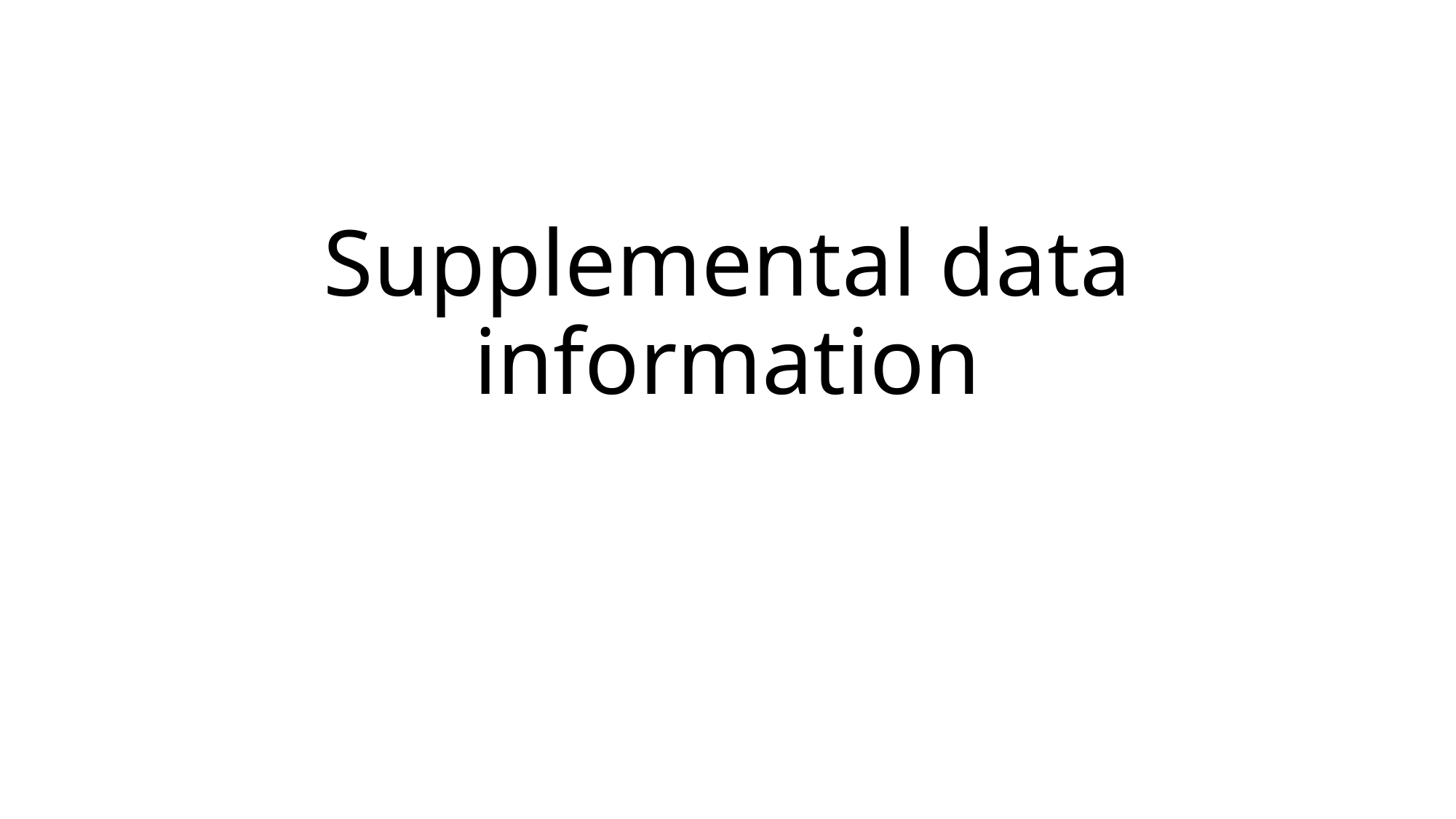

# Supplemental data information

## Slide 2
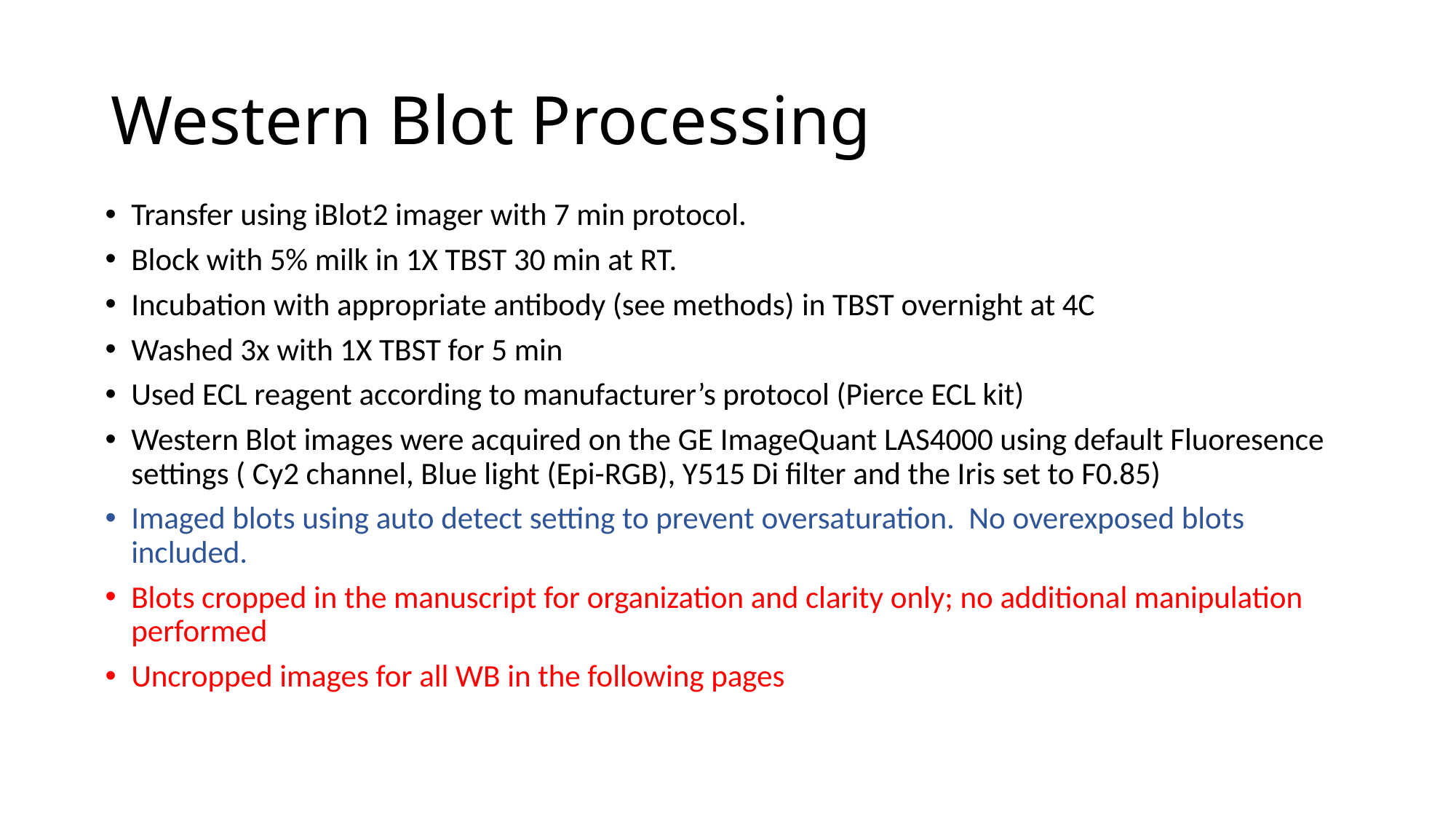

# Western Blot Processing
Transfer using iBlot2 imager with 7 min protocol.
Block with 5% milk in 1X TBST 30 min at RT.
Incubation with appropriate antibody (see methods) in TBST overnight at 4C
Washed 3x with 1X TBST for 5 min
Used ECL reagent according to manufacturer’s protocol (Pierce ECL kit)
Western Blot images were acquired on the GE ImageQuant LAS4000 using default Fluoresence settings ( Cy2 channel, Blue light (Epi-RGB), Y515 Di filter and the Iris set to F0.85)
Imaged blots using auto detect setting to prevent oversaturation. No overexposed blots included.
Blots cropped in the manuscript for organization and clarity only; no additional manipulation performed
Uncropped images for all WB in the following pages

## Slide 3
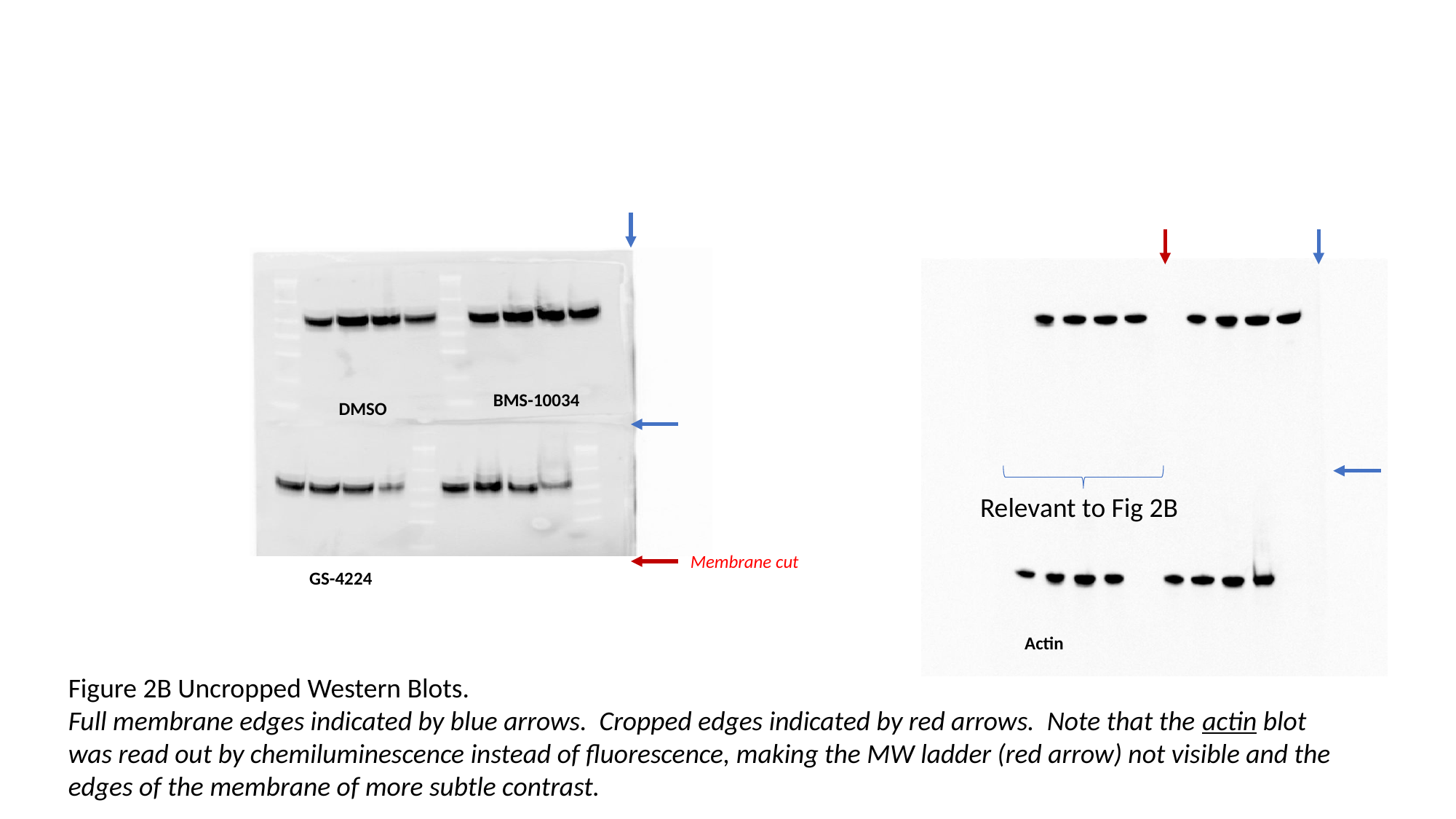

#
BMS-10034
DMSO
Relevant to Fig 2B
Membrane cut
GS-4224
Actin
Figure 2B Uncropped Western Blots.
Full membrane edges indicated by blue arrows. Cropped edges indicated by red arrows. Note that the actin blot was read out by chemiluminescence instead of fluorescence, making the MW ladder (red arrow) not visible and the edges of the membrane of more subtle contrast.

## Slide 4
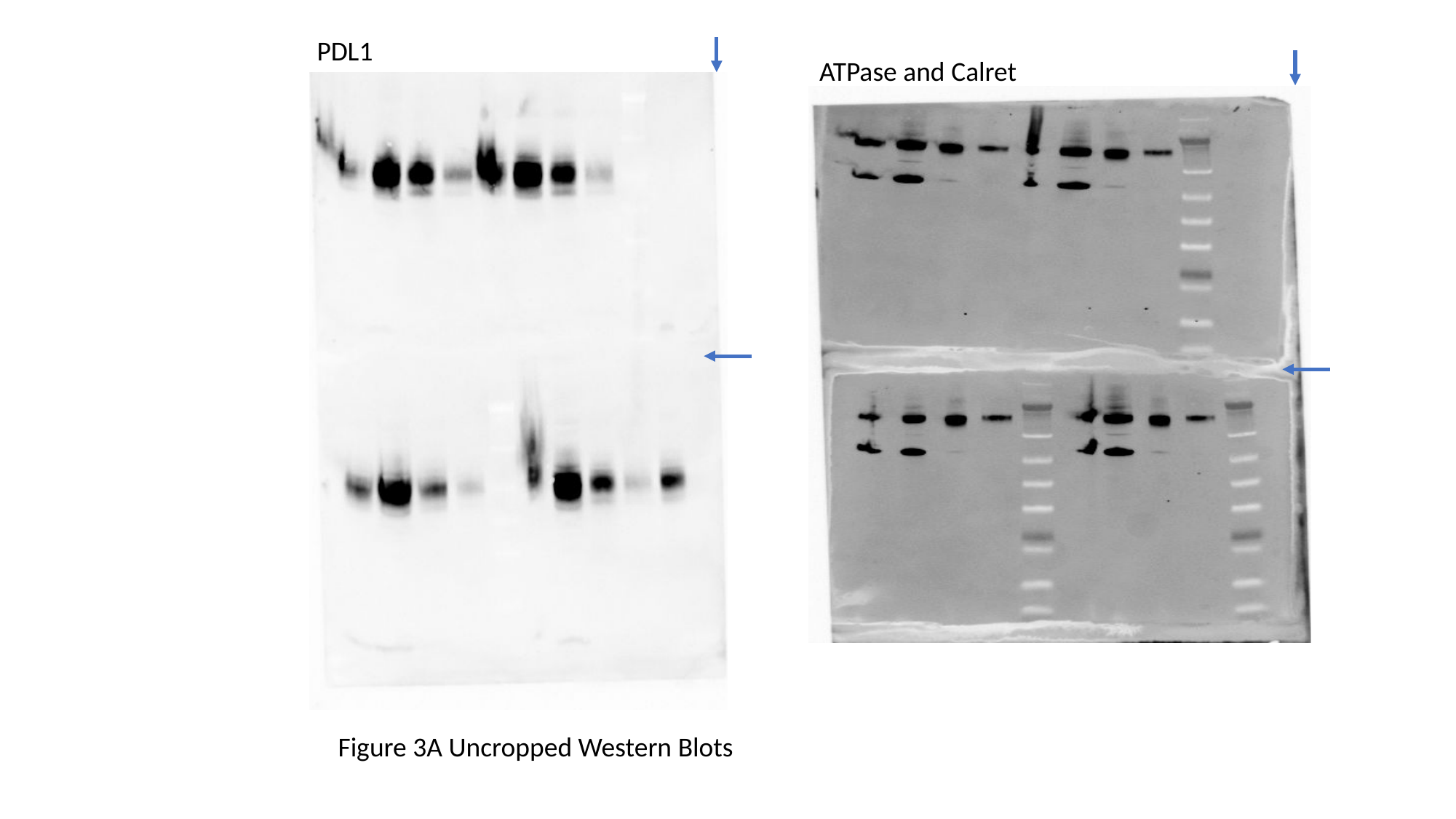

PDL1
ATPase and Calret
Figure 3A Uncropped Western Blots

## Slide 5
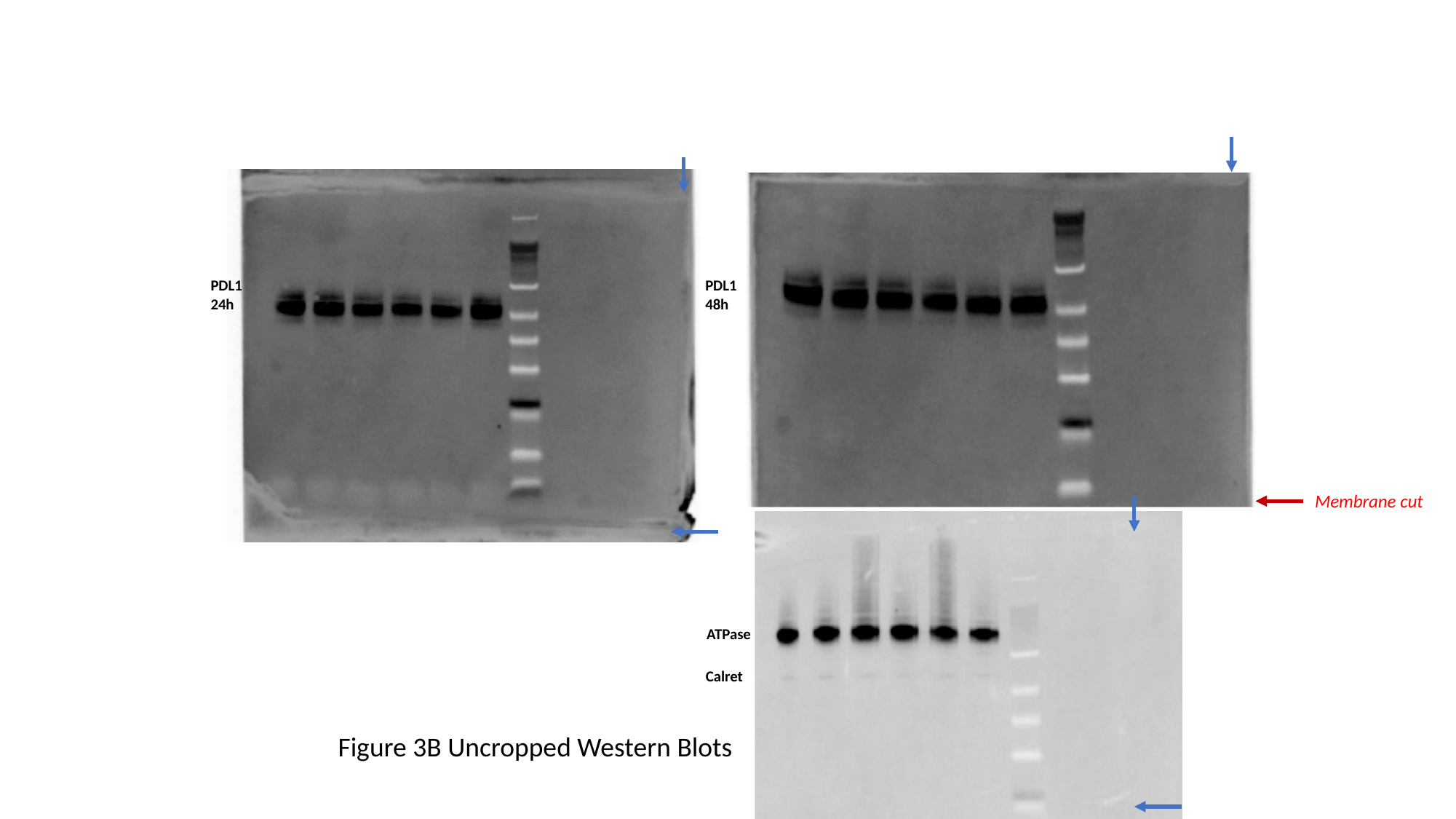

#
PDL1
24h
PDL1
48h
Membrane cut
ATPase
Calret
Figure 3B Uncropped Western Blots

## Slide 6
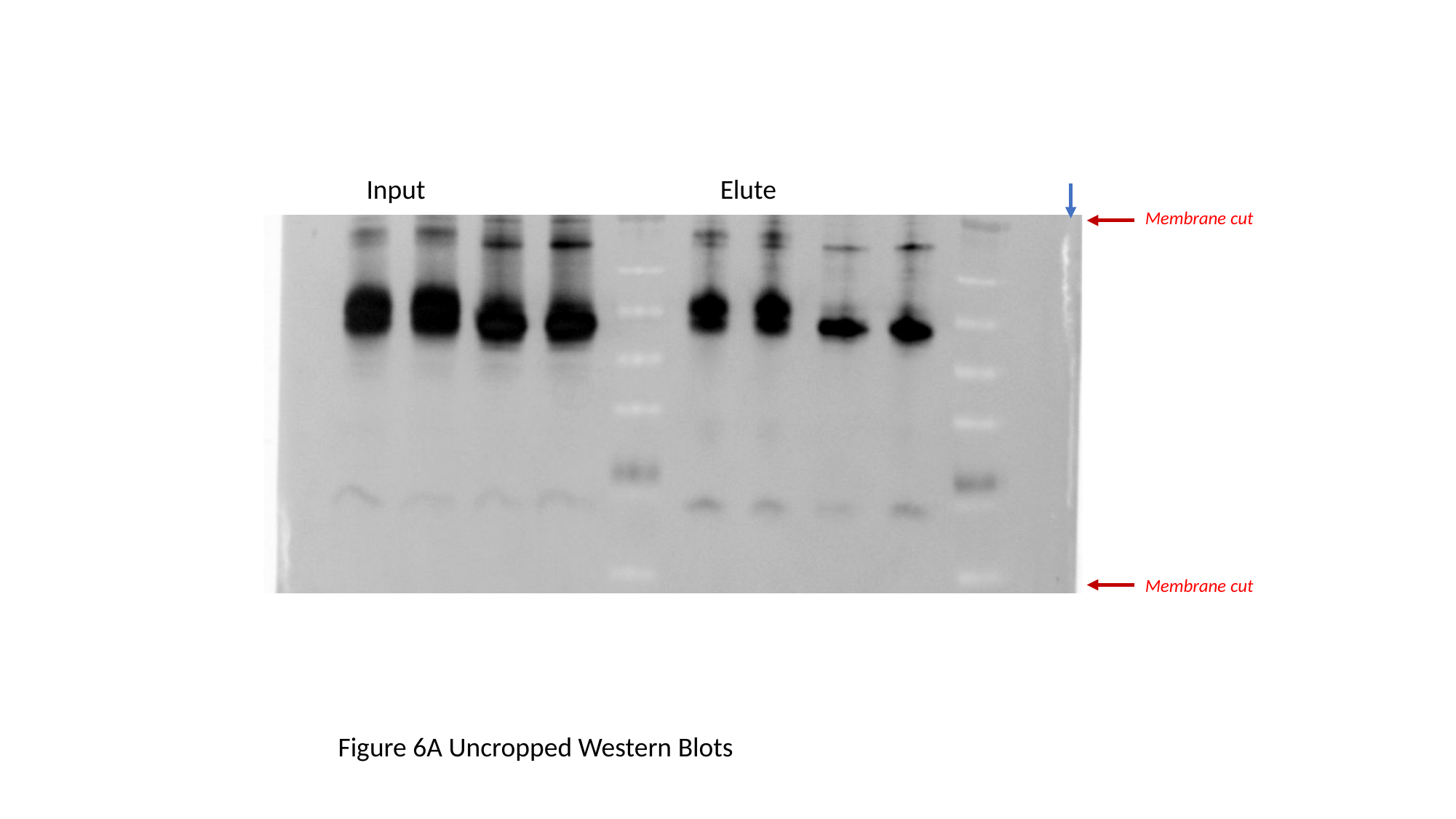

Input
Elute
Membrane cut
Membrane cut
Figure 6A Uncropped Western Blots

## Slide 7
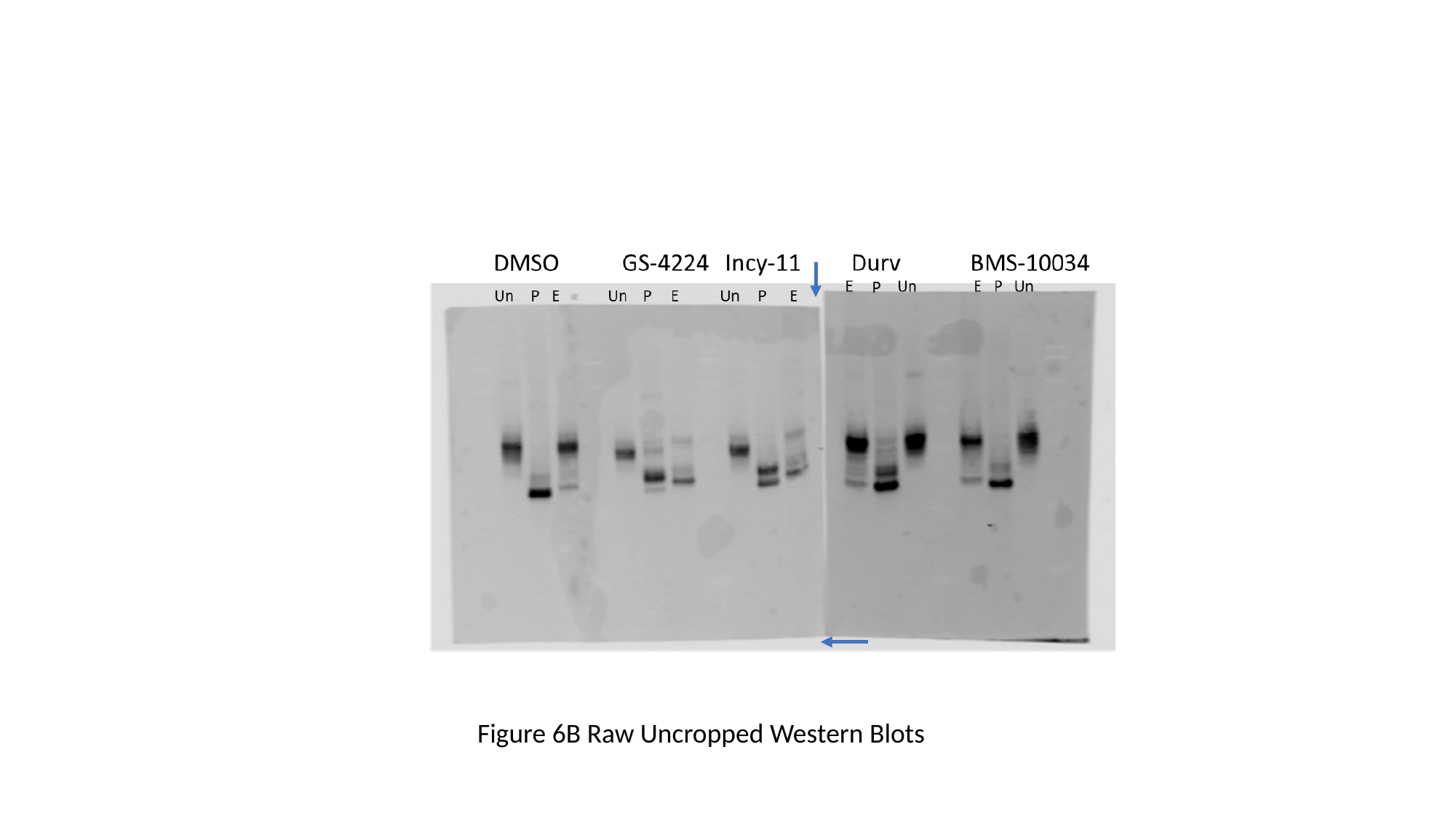

#
Figure 6B Raw Uncropped Western Blots

## Slide 8
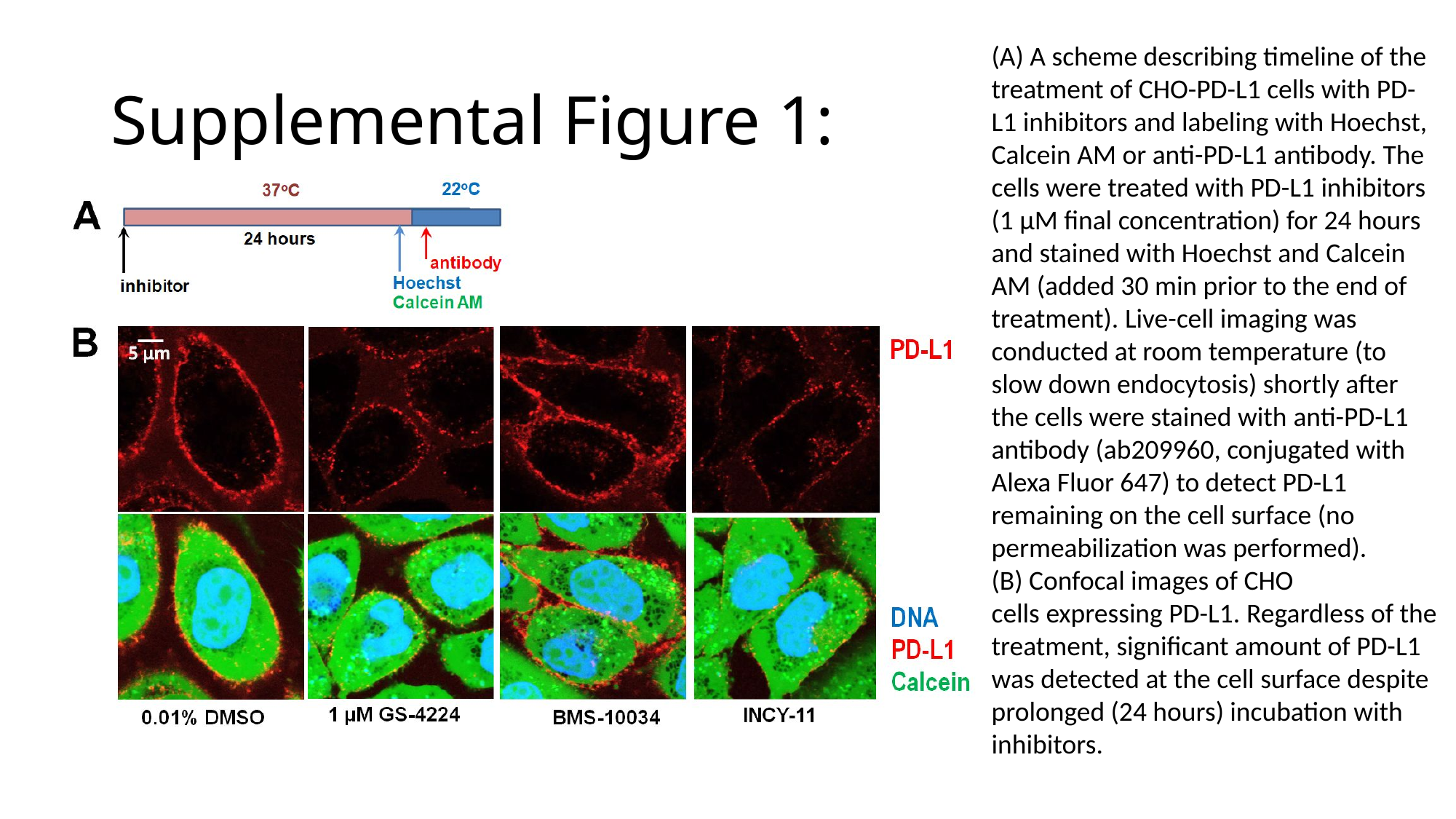

(A) A scheme describing timeline of the treatment of CHO-PD-L1 cells with PD-L1 inhibitors and labeling with Hoechst, Calcein AM or anti-PD-L1 antibody. The cells were treated with PD-L1 inhibitors (1 µM final concentration) for 24 hours and stained with Hoechst and Calcein AM (added 30 min prior to the end of treatment). Live-cell imaging was conducted at room temperature (to slow down endocytosis) shortly after the cells were stained with anti-PD-L1 antibody (ab209960, conjugated with Alexa Fluor 647) to detect PD-L1 remaining on the cell surface (no permeabilization was performed).
(B) Confocal images of CHO cells expressing PD-L1. Regardless of the treatment, significant amount of PD-L1 was detected at the cell surface despite prolonged (24 hours) incubation with inhibitors.
# Supplemental Figure 1:

## Slide 9
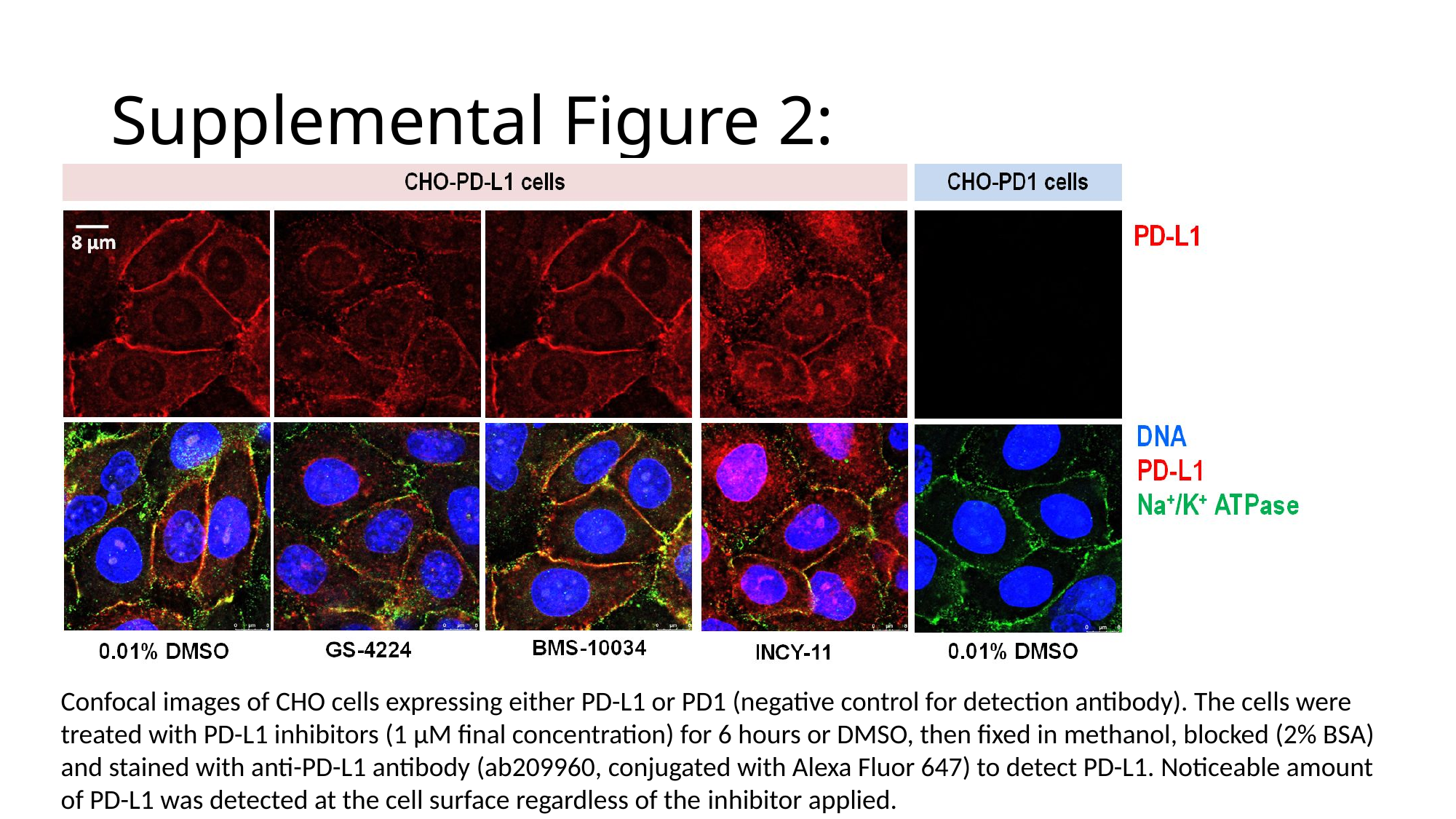

# Supplemental Figure 2:
Confocal images of CHO cells expressing either PD-L1 or PD1 (negative control for detection antibody). The cells were treated with PD-L1 inhibitors (1 µM final concentration) for 6 hours or DMSO, then fixed in methanol, blocked (2% BSA)  and stained with anti-PD-L1 antibody (ab209960, conjugated with Alexa Fluor 647) to detect PD-L1. Noticeable amount of PD-L1 was detected at the cell surface regardless of the inhibitor applied.

## Slide 10
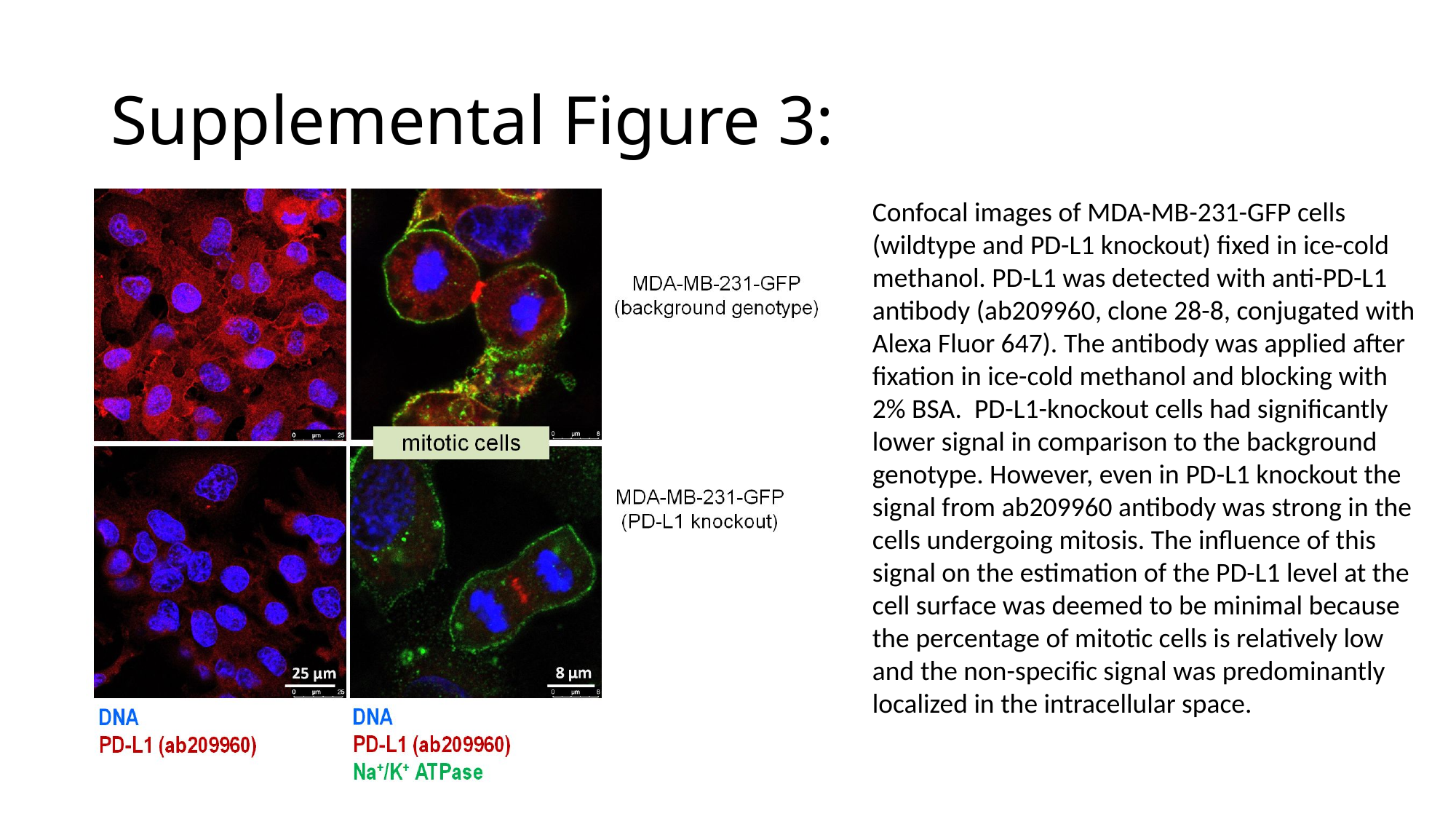

# Supplemental Figure 3:
Confocal images of MDA-MB-231-GFP cells (wildtype and PD-L1 knockout) fixed in ice-cold methanol. PD-L1 was detected with anti-PD-L1 antibody (ab209960, clone 28-8, conjugated with Alexa Fluor 647). The antibody was applied after fixation in ice-cold methanol and blocking with 2% BSA.  PD-L1-knockout cells had significantly lower signal in comparison to the background genotype. However, even in PD-L1 knockout the signal from ab209960 antibody was strong in the cells undergoing mitosis. The influence of this signal on the estimation of the PD-L1 level at the cell surface was deemed to be minimal because the percentage of mitotic cells is relatively low and the non-specific signal was predominantly localized in the intracellular space.

## Slide 11
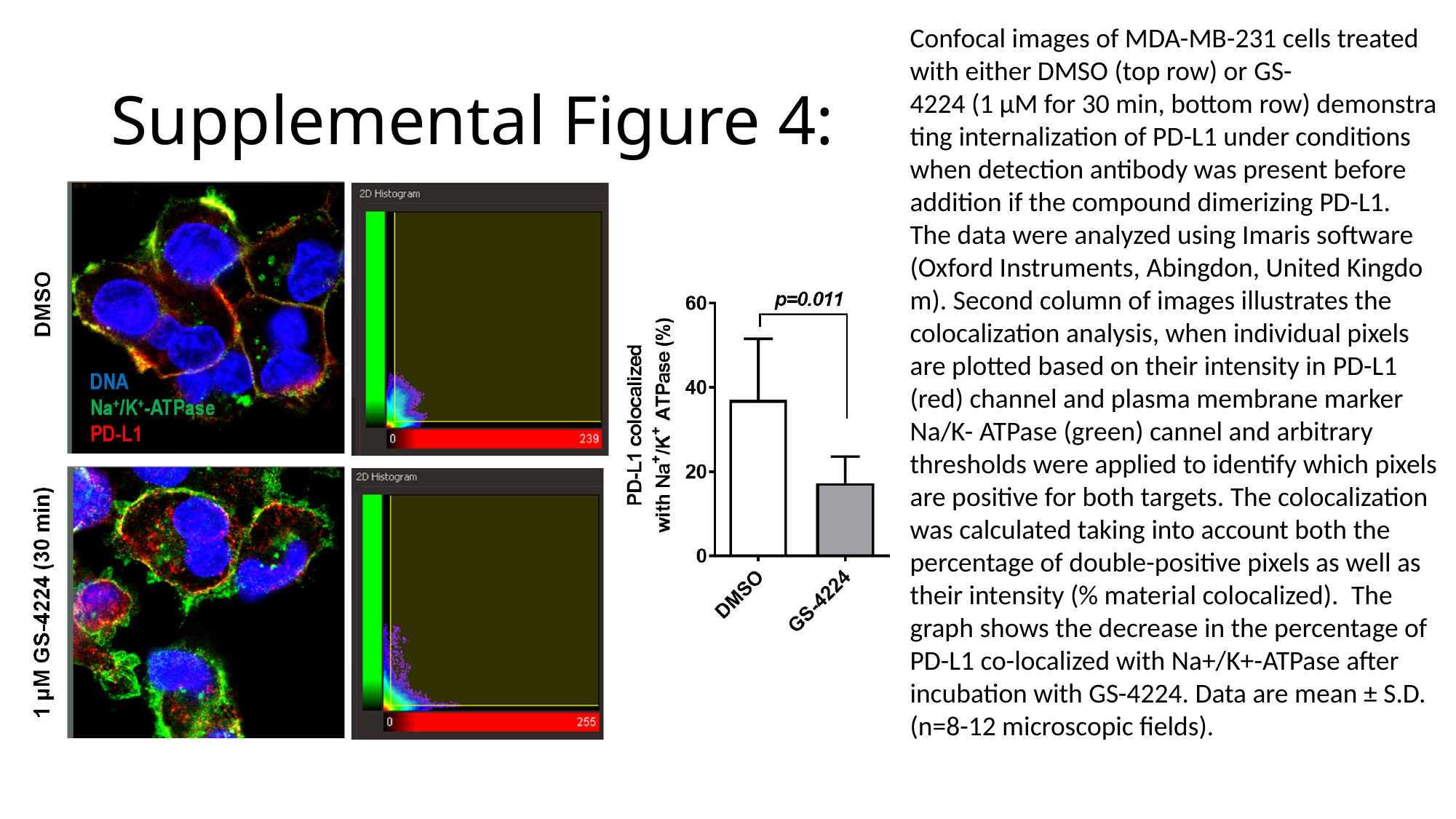

Confocal images of MDA-MB-231 cells treated with either DMSO (top row) or GS-4224 (1 µM for 30 min, bottom row) demonstrating internalization of PD-L1 under conditions when detection antibody was present before addition if the compound dimerizing PD-L1.  The data were analyzed using Imaris software (Oxford Instruments, Abingdon, United Kingdom). Second column of images illustrates the colocalization analysis, when individual pixels are plotted based on their intensity in PD-L1 (red) channel and plasma membrane marker Na/K- ATPase (green) cannel and arbitrary thresholds were applied to identify which pixels are positive for both targets. The colocalization was calculated taking into account both the percentage of double-positive pixels as well as their intensity (% material colocalized).  The graph shows the decrease in the percentage of PD-L1 co-localized with Na+/K+-ATPase after  incubation with GS-4224. Data are mean ± S.D. (n=8-12 microscopic fields).
# Supplemental Figure 4:

## Slide 12
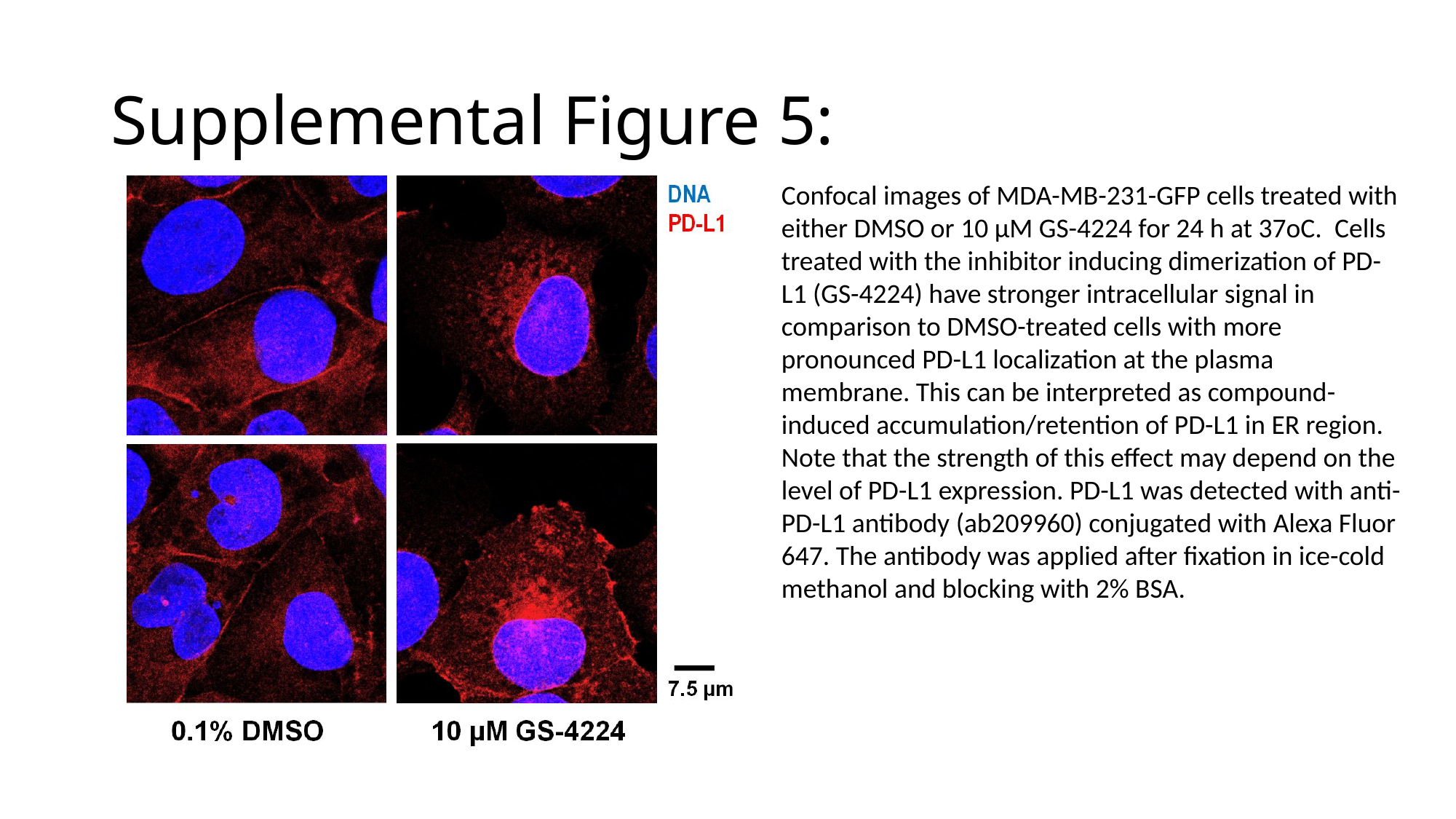

# Supplemental Figure 5:
Confocal images of MDA-MB-231-GFP cells treated with either DMSO or 10 µM GS-4224 for 24 h at 37oC.  Cells treated with the inhibitor inducing dimerization of PD-L1 (GS-4224) have stronger intracellular signal in comparison to DMSO-treated cells with more pronounced PD-L1 localization at the plasma membrane. This can be interpreted as compound-induced accumulation/retention of PD-L1 in ER region.  Note that the strength of this effect may depend on the level of PD-L1 expression. PD-L1 was detected with anti-PD-L1 antibody (ab209960) conjugated with Alexa Fluor 647. The antibody was applied after fixation in ice-cold methanol and blocking with 2% BSA.

## Slide 13
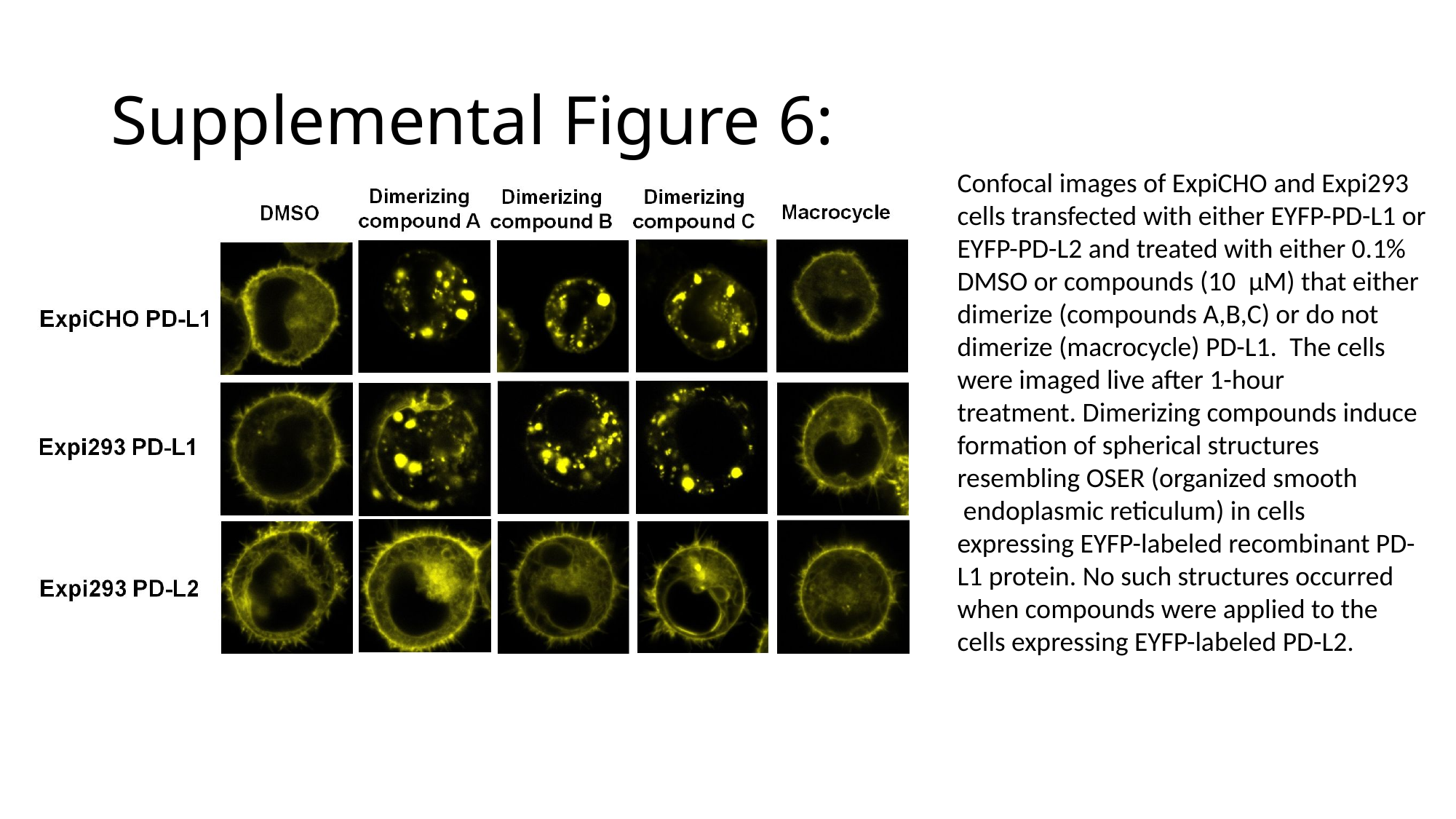

# Supplemental Figure 6:
Confocal images of ExpiCHO and Expi293 cells transfected with either EYFP-PD-L1 or EYFP-PD-L2 and treated with either 0.1% DMSO or compounds (10  µM) that either dimerize (compounds A,B,C) or do not dimerize (macrocycle) PD-L1.  The cells were imaged live after 1-hour treatment. Dimerizing compounds induce formation of spherical structures resembling OSER (organized smooth  endoplasmic reticulum) in cells expressing EYFP-labeled recombinant PD-L1 protein. No such structures occurred when compounds were applied to the cells expressing EYFP-labeled PD-L2.

## Slide 14
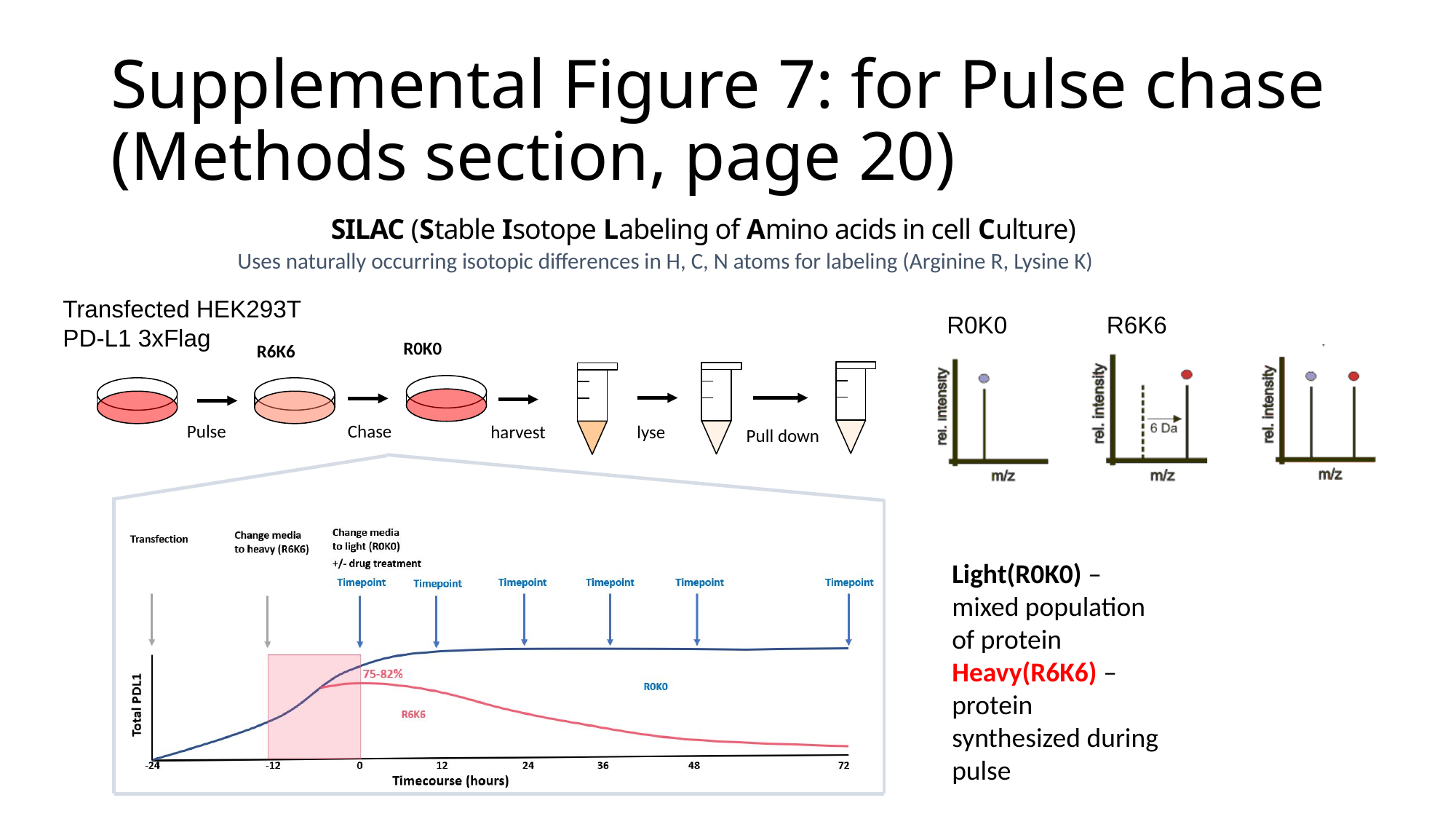

# Supplemental Figure 7: for Pulse chase (Methods section, page 20)
SILAC (Stable Isotope Labeling of Amino acids in cell Culture)
Uses naturally occurring isotopic differences in H, C, N atoms for labeling (Arginine R, Lysine K)
Transfected HEK293T
PD-L1 3xFlag
R6K6
R0K0
R0K0
R6K6
Pulse
Chase
harvest
lyse
Pull down
Light(R0K0) – mixed population of protein
Heavy(R6K6) – protein synthesized during pulse
